# Up-regulation of microRNA-203 in influenza A virus infection inhibits viral replication by targeting DR1

**DOI:** 10.1038/s41598-018-25073-9

**Published:** 2018-05-01

**Authors:** Sen Zhang, Jing Li, Junfeng Li, Yinhui Yang, Xiaoping Kang, Yuchang Li, Xiaoyan Wu, Qingyu Zhu, Yusen Zhou, Yi Hu

**Affiliations:** State Key Laboratory of Pathogen and Biosecurity, Beijing Institute of Microbiology and Epidemiology, Beijing, 100071 People’s Republic of China

## Abstract

MicroRNAs (miRNAs) are small noncoding RNA molecules that play important roles in various biological processes. Much evidence shows that miRNAs are closely associated with numerous virus infections; however, involvement of cellular miRNAs in influenza A virus (IAV) infection is unclear. Here, we found that expression of miR-203 was up-regulated markedly via two different mechanisms during IAV infection. First, we examined the effects of type I interferon induced by IAV on direct activation of miR-203 expression. Next, we showed that DNA demethylation within the miR-203 promoter region in A549 cells induced its up-regulation, and that expression of DNA methyltransferase 1 was down-regulated following H5N1 virus infection. Ectopic expression of miR-203 in turn inhibited H5N1 virus replication by targeting down-regulator of transcription 1 (DR1), which was identified as a novel target of miR-203. Silencing DR1 in miR-203 knockout cells using a specific siRNA inhibited replication of the H5N1 virus, an effect similar to that of miR-203. In summary, the data show that host cell expression of miR-203 is up-regulated upon IAV infection, which increases antiviral responses by suppressing a novel target gene, DR1. Thus, we have identified a novel mechanism underlying the relationship between miR-203 and IAV infection.

## Introduction

MicroRNAs (miRNAs) are post-transcriptional regulators that play important roles in a number of biological processes, including cell proliferation, differentiation, apoptosis, stress responses, and regulation of inflammatory pathways^1^. Altered expression of some miRNAs is linked to diseases such as cancer, metabolic disorders, and infarction^2^. Meanwhile, miRNAs are also attracting attention as significant players during viral infection. A number of DNA viruses encode their own miRNAs, which are ideal tools for establishing an intracellular environment conducive to viral replication^3^. Compared with viral proteins, miRNAs have an advantage in that they are non-immunogenic, target mRNAs more accurately, and can evolve rapidly to target new transcripts^4,5^. To date, more than 400 viral miRNAs have been identified, mainly in herpesviruses, adenoviruses, ascoviruses, and polyomaviruses^6–9^. In addition to viral-encoded miRNAs, expression of host cellular miRNAs can also be influenced markedly during virus infections. Alteration of cellular miRNAs can have two different results: viruses change the intracellular environment to evade antiviral immune responses, or host cells trigger antiviral defenses that affect viral replication^10^.

Influenza A virus (IAV), a negative single-stranded RNA virus belonging to the family Orthomyxoviridae, causes a contagious respiratory infection with symptoms such as chills, headache, fever, and general pain; in some severe cases, infection can prove fatal^11^. Once the infection is initiated, the virus utilizes the host cellular machinery to facilitate replication and evasion of antiviral immune responses^12^; however, host cells will not easily accept viral invasion and they trigger a series of antiviral responses^13^. In the “fight” between IAVs and host cells, miRNAs play a necessary and regulatory role. Studies show that expression of more than 100 host miRNAs is altered during infection; these miRNAs promote or inhibit viral replication^14,15^. Some host miRNAs target IAV genes directly to inhibit replication. For instance, cellular miR-584-5p and miR-1249 are down-regulated upon H5N1 virus infection; these miRNAs target the PB2 gene to inhibit viral replication^16^. Some other host miRNAs regulate virus infection by participating in certain intracellular signaling pathways. For example, cellular miR-144 suppresses host immune responses to IAVs by silencing the TRAF6-IRF7 signaling axis^17^, while up-regulated cellular miR-136, which is a suppressor of IAV replication, can act as a ligand for RIG-1 to increase innate immunity^18^. However, the roles of miRNAs in regulating host-virus interactions remain largely unknown. Considering the numerous molecular targets of miRNAs and the increasing evidence of a connection with viral infections, it seems that miRNAs have great potential as biomarkers for the diagnosis and treatment of clinical diseases. Identifying the mechanisms underlying miRNA-mediated regulation of virus infection would help to identify novel targets for antiviral agents.

Here, we aimed to further identify candidate miRNAs that participate in host immune responses to IAV infection. First, we examined changes in the abundance of miRNAs in A549 cells either mock-infected or infected with IAV. Differentially expressed miRNAs were identified, the most prominent being miR-203. MiR-203 is a miRNA abundant in skin where it promotes epidermal differentiation by repressing proliferative potential^19^. Moreover, expression of miR-203 is down-regulated in hepatocellular carcinoma caused by hepatitis C virus, and in cervical carcinoma caused by human papillomaviruses^20,21^, confirming that miR-203 functions as a tumor suppressor. In addition, others have examined the role of miR-203 in other viral infections, including coxsackievirus B3, hepatitis B virus, and Sendai virus^22–24^. However, no studies have examined the relationship between IAV infection and miR-203 expression in detail; only Buggele *et al*. (who conducted research on Sendai virus and miR-203) reported that miR-203 could be induced in A549 cells infected with A/Udorn/72 and A/WSN/33 strains of IAV^24^, but further investigation was not provided. Here, we found that up-regulated expression of miR-203 was positively correlated with increased type I interferon (IFN) responses and DNA demethylation caused by IAV. MiR-203 inhibited IAV replication by silencing expression of down-regulator of transcription 1 (DR1), an as yet unidentified target of miR-203. The data presented herein increase our understanding of the role of host miRNA in the stress response to IAV infection.

## Results

### IAV infection of A549 cells up-regulates expression of miR-203

**Figure 1:**
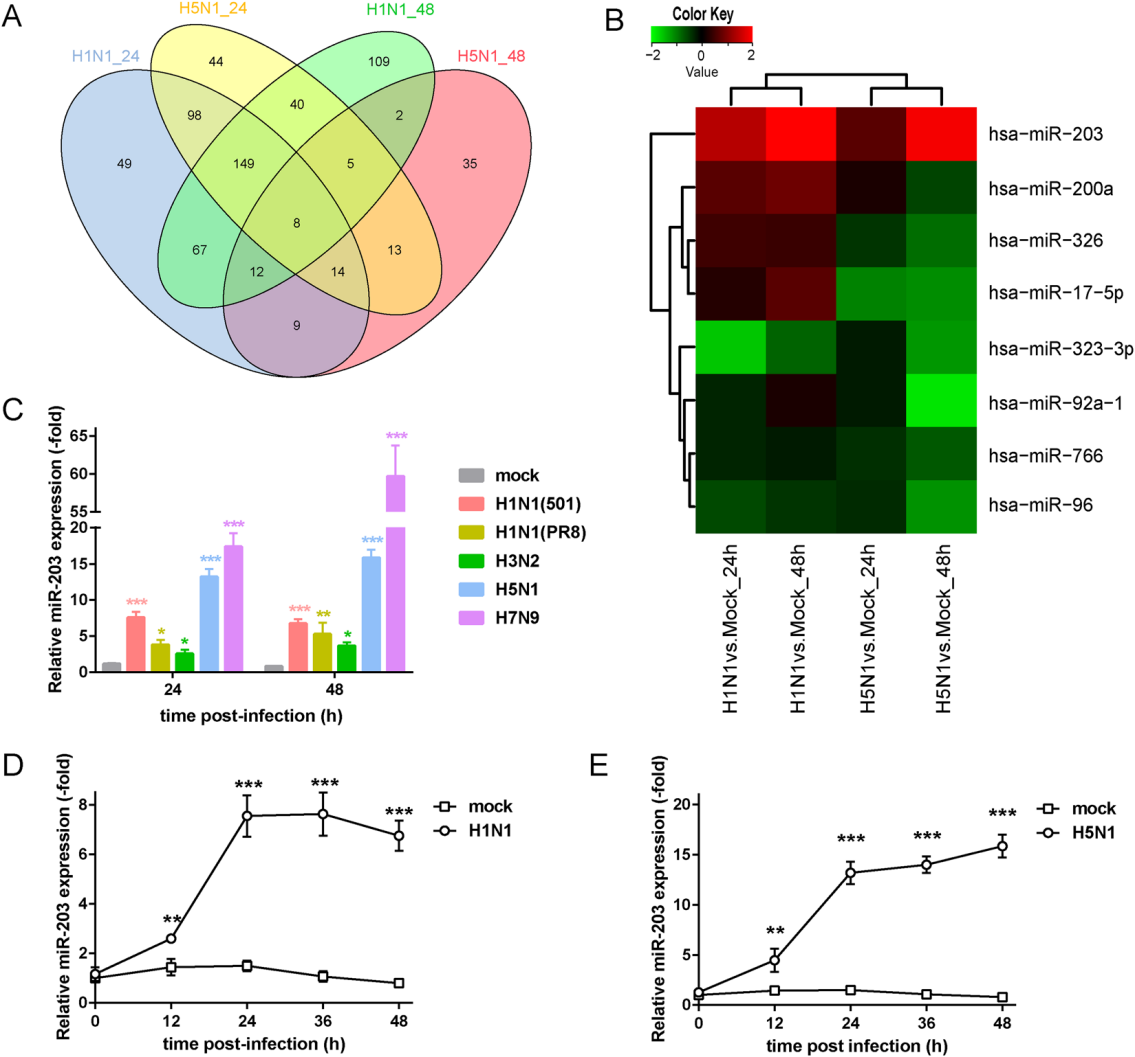
Influenza A virus (IAV) infection of A549 cells up-regulates expression of miR-203. (**A**) Venn diagram illustrates the numbers of miRNAs identified in each group and the number of common miRNAs within the four groups. (**B**) Heatmap of miRNA expression in A549 cells infected with H1N1 (501) and H5N1 viruses for 24 or 48 h. Each value for the virus/mock average signal ratio was expressed as log10 when drawing the Heatmap. Red denotes up-regulation, while green denotes down-regulation. (**C**) A549 cells were mock-infected or infected with H1N1 (501), H1N1 (PR8), H3N2, H5N1, or H7N9 for 24 or 48 h, and abundance of miR-203 was assessed by quantitative real-time PCR (qPCR). (**D** and **E**) A549 cells were mock-infected or infected with H1N1 (501) (**D**) or H5N1 viruses (**E**). Cells were harvested at different times (12, 24, 36, and 48 h), and dynamic changes in miR-203 expression were assessed by qPCR. Data are expressed as the mean ± SD of three independent experiments. *p < 0.05; **p < 0.01; and ***p < 0.001 (Student’s *t* test).

To investigate whether IAV infection alters cellular miRNA profiles, A549 cells were either mock-infected or infected with two different subtypes of IAV, A/Beijing/501/2009 (H1N1; multiplicity of infection (MOI) = 5) and A/Vietnam/1194/2004 (H5N1; MOI = 2), for 24 (H1N1_24 group and H5N1_24 group) or 48 h (H1N1_48 group and H5N1_48 group). Total RNA was purified from cell samples, and microarray analysis of miRNAs was performed. Scatter diagrams (Supplementary Fig. S1) showed the expression level of four groups of miRNAs. Differentially expressed miRNAs were screened out according to the following criteria: a detection *p* value < 0.01 and a fold change in expression ≥ 2. In accordance with these standards, 406 differentially expressed miRNAs were selected from the H1N1_24 group (315 down-regulated and 91 up-regulated); 371 miRNAs were selected from the H1N1_48 group (180 down-regulated and 191 up-regulated); 70 miRNAs were selected from the H5N1_24 group (43 down-regulated and 27 up-regulated); and 392 miRNAs were selected from the H5N1_48 group (297 down-regulated and 95 up-regulated). Comparison of all these differentially expressed miRNAs revealed that each group contained eight common miRNAs (Fig. 1A), namely miR-203, miR-200a, miR-326, miR-17-5p, miR-323-3p, miR-92a-1, miR-766, and miR-96. The Heatmap revealed alterations in the abundance of these eight miRNAs upon infection with H1N1 and H5N1; of these, only miR-203 was up-regulated upon infection by both viruses (Fig. 1B). Next, A549 cells were infected with different subtypes of IAV (H1N1 (501), MOI = 5; H1N1 (PR8), MOI = 5; H3N2, MOI = 5; H5N1, MOI = 2; and H7N9, MOI = 5) for 24 or 48 h. We then measured alterations in expression of miR-203 by quantitative real-time PCR (qPCR). The results revealed clear up-regulation of miR-203 expression in response to all these viruses (Fig. 1C). We chose to use the typical highly pathogenic avian influenza virus, H5N1, and the pandemic H1N1 influenza virus for further analysis of dynamic changes in miR-203, and confirmed that IAVs induce expression of miR-203 in A549 cells (Fig. 1D and E).

### Type I IFN participates in transcriptional regulation of miR-203 to directly induce its expression

**Figure 2:**
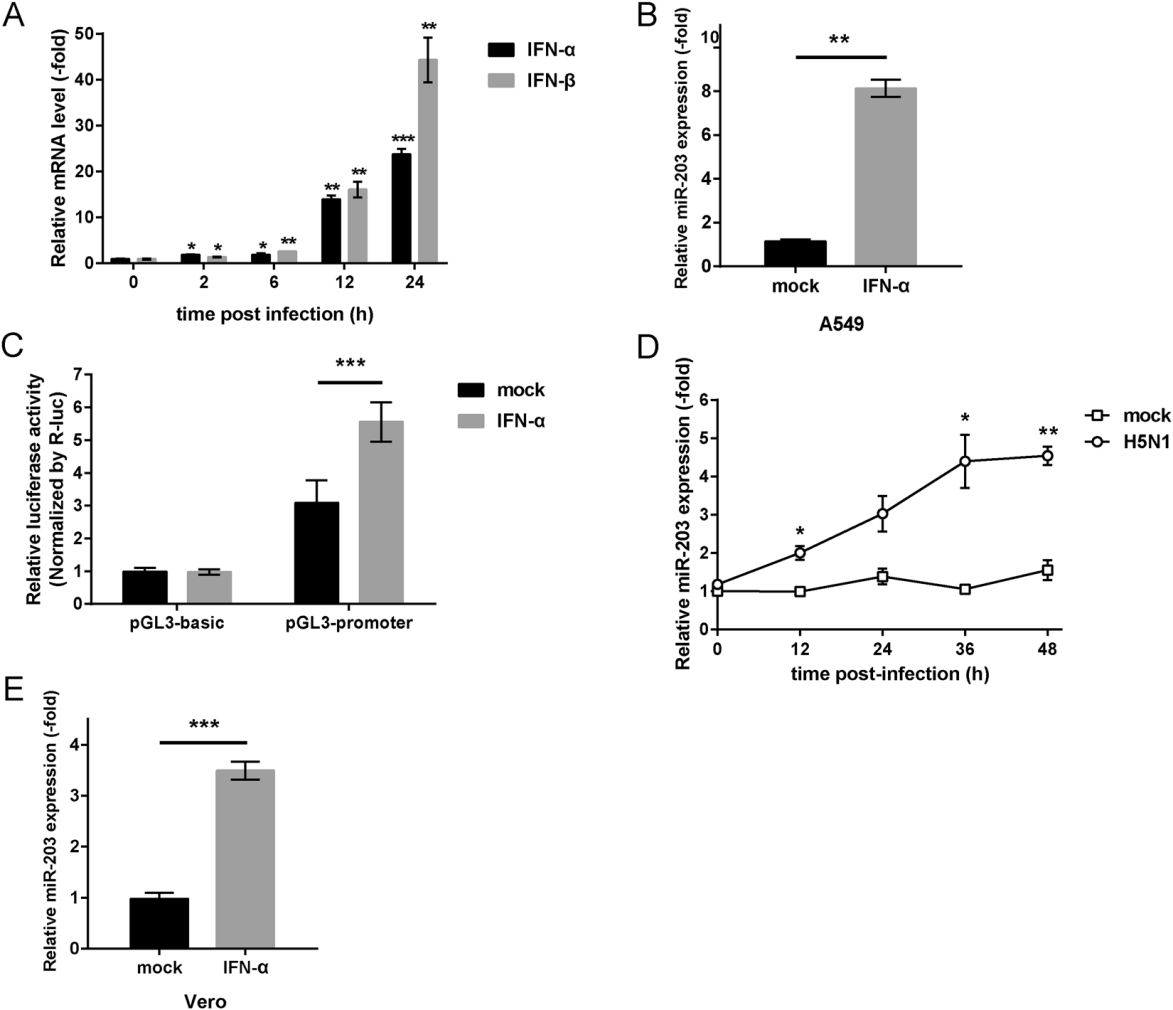
Type I interferon (IFN) participates in transcriptional regulation of miR-203 to promote its expression directly. (**A**) A549 cells were infected with H5N1 (MOI = 2) viruses and harvested at different times (2, 6, 12, and 24 h). The abundance of IFN-α and IFN-β mRNA was measured by quantitative real-time PCR (qPCR). (**B** and **C**) A549 cells (**B**) and Vero cells (**C**) were treated with IFN-α (2000 units/ml) for 12 h, and expression of miR-203 was measured by qPCR. (D) A549 cells were co-transfected for 24 h with luciferase reporter vectors (pGL3-promoter) and a pRL-TK plasmid (pGL3-basic vectors were used as a negative control). Then, cells were treated with IFN-α for another 12 h followed by measurement of luciferase activity in a dual-luciferase assay. The results are presented as the normalized ratio of Firefly to Renilla luciferase activity. (**E**) Vero cells were mock-infected or infected with H5N1 (MOI = 2) viruses. Cells were harvested at different times (12, 24, 36, and 48 h), and abundance of miR-203 was measured by qPCR. Data are expressed as the mean + SD of three independent experiments. *p < 0.05; **p < 0.01; and ***p < 0.001 (Student’s *t* test).

IFNs are an important component of host innate immune responses and are activated rapidly during IAV infection^25^. The main IFNs generated during IAV infection are type I IFN (IFN-α/β) and type III IFN (IFN-λ); the former is induced more strongly and more quickly than the latter^26^. We found that both IFN-α and IFN-β mRNA were induced strongly in A549 cells at 12 h post-infection with H5N1 (MOI = 2) viruses (Fig. 2A). Production and secretion of IFNs is implicated in regulation of miRNAs^27^, and IFN-α induces miR-203 expression directly during Sendai virus infection^24^. To determine the role of type I IFN in up-regulating miR-203 during IAV infection, we performed the experiments as done in the previous study of Buggele *et al*.^24^; to this end, we treated A549 cells for 12 h with IFN-α alone (2,000 units/ml) and then extracted total RNA to analyze miR-203 levels. QPCR results indicated that type I IFN induced miR-203 expression directly (Fig. 2B), a result consistent with that of Buggele *et al*.^24^. Next, the 2500 bp promoter region of miR-203 was amplified and cloned into the pGL3-basic vector to construct a luciferase reporter system (pGL3-promoter). A dual-luciferase assay was then conducted in A549 cells co-transfected with luciferase reporter vectors (pGL3-promoter) and a pRL-TK plasmid, with pGL3-basic vectors (empty vectors lacking the promoter region) as a negative control. Measurement of luciferase activity revealed that IFN-α stimulated miR-203 promoter activity (Fig. 2C). Thus, type I IFN induces expression of miR-203 at the transcriptional level upon IAV infection.

To explore whether type I IFN is essential for induction of miR-203 during IAV infection, Vero cells (a cell line defective in IFNs production)^28,29^, were mock-infected or infected with H5N1 (MOI = 2) viruses. Expression of miR-203 was also increased when compared with that in the mock group (Fig. 2D), although the fold of increase was smaller than that in A549 cells. This result was different from the previous finding using Sendai virus, which induced little miR-203 expression in Vero cells^24^, indicating that factors other than type I IFN were also involved in induction of miR-203 during IAV infection. Next, to further confirm the function of type I IFN, a similar experiment was performed as done in the study of Buggele *et al*.^24^. Vero cells were treated for 12 h with exogenous IFN-α (2,000 units/ml). The results showed that miR-203 was also up-regulated (Fig. 2E), a result consistent with that observed in A549 cells (Fig. 2B); this was further confirmation that type I IFN plays an important role in inducing miR-203.

### The demethylation of the miR-203 promoter region caused by IAV up-regulates miR-203 expression

**Figure 3:**
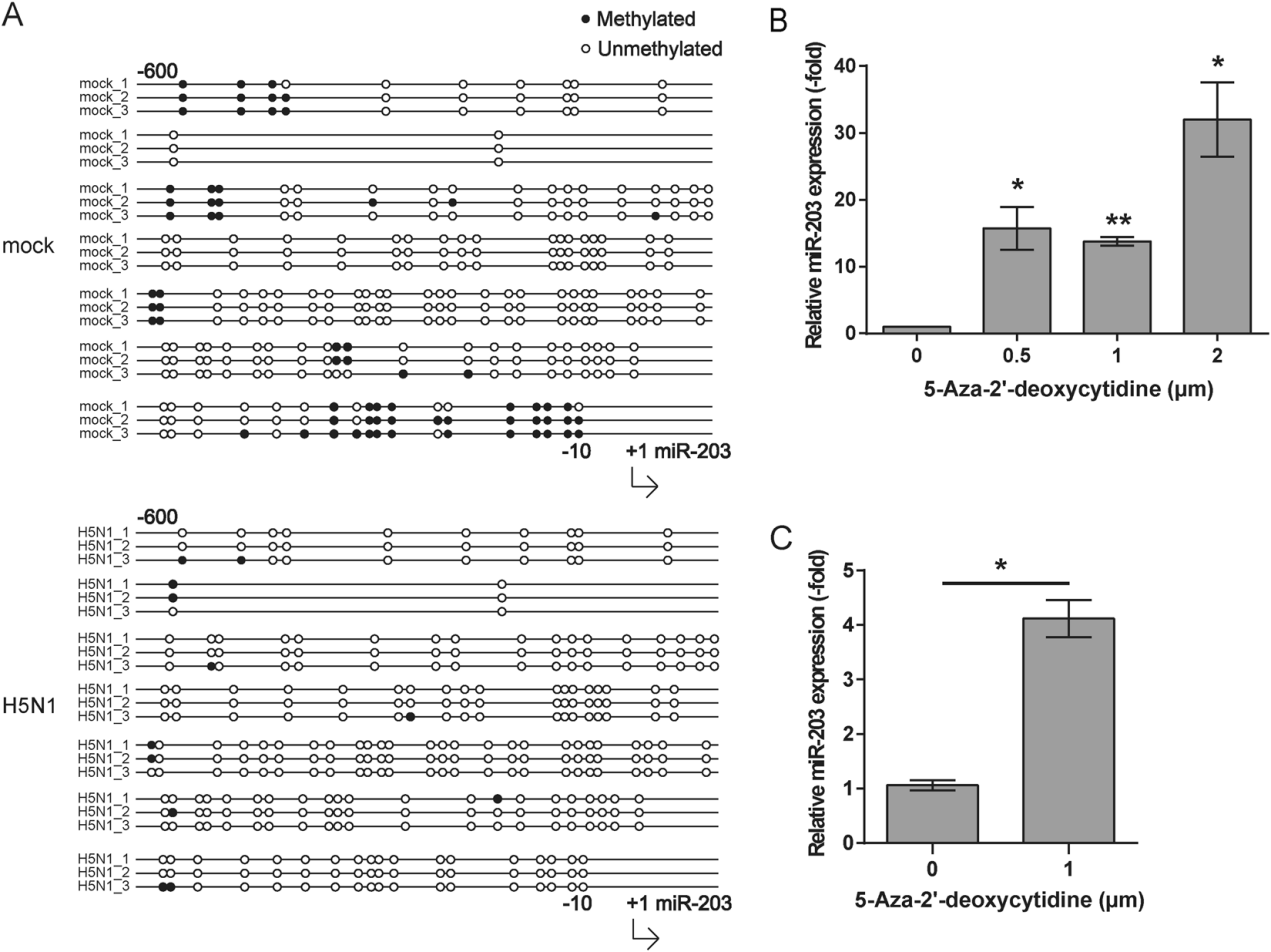
IAV-mediated demethylation of the miR-203 promoter region up-regulates expression of miR-203. (**A**) Bisulfite sequencing PCR of the miR-203 promoter region was performed in A549 cells mock-infected or infected with H5N1 (MOI = 2) viruses for 24 h. The sequenced region covered −600 to −10 bp within the miR-203 promoter region. Three lines denote three replicates in per group. Open circles, unmethylated; solid circles, methylated. (**B**) A549 cells were treated for 48 h with 5-aza-2′-deoxycytidine, a methylation inhibitor, at a final concentration of 0.5, 1, or 2 μM, and total RNA was purified to analyze abundance of miR-203 by quantitative real-time PCR (qPCR). (**C**) Vero cells were treated for 48 h with 5-aza-2′-deoxycytidine (final concentration, 1 μM) and total RNA purified. The abundance of miR-203 was measured by qPCR. Data are expressed as the mean + SD of three independent experiments. *p < 0.05; **p < 0.01; and ***p < 0.001 (Student’s *t* test).

Epigenetic modifications have been reported to play a pivotal role in miR-203 expression in the tumorigenesis and tumor progression^30–33^. To dissect whether this mechanism was also involved in IAV induced miR-203 expression, we performed bisulfite sequencing PCR of mock-infected or H5N1 (MOI = 2) virus-infected A549 cells to examine epigenetic changes (e.g., DNA methylation modifications) induced by viral infection in miR-203 promoter region. As shown in Fig. 3A, the CpG island in the miR-203 promoter (which spans the region from −600 to −10 bp) in infected A549 cells carried fewer methylations than that in mock-infected cells (other bisulfite sequencing results are not shown). This suggests that IAV infection triggers demethylation of the promoter region. To further explore the relationship between DNA demethylation and up-regulation of miR-203, A549 cells were treated for 48 h with 5-aza-2′-deoxycytidine, a methylation inhibitor, at a final concentration of 0.5, 1, or 2 μM and total RNA was purified to analyze the abundance of miR-203. The results showed that 5-aza-2′-deoxycytidine stimulated production of miR-203 in a dose-dependent manner (Fig. 3B). Thus, the data suggest that up-regulated miR-203 expression is associated with DNA demethylation of the promoter region caused by IAV infection. Next, Vero cells were also treated for 48 h with 5-aza-2′-deoxycytidine at a concentration of 1 μM; the results revealed similar up-regulation of miR-203 (Fig. 3C). This indicates that the effect of DNA demethylation in Vero cells correlates positively with expression of miR-203. This may be a key factor for induction of miR-203 in IAV-infected Vero cells.

### Down-regulation of DNMT1 induces miR-203 expression upon IAV infection

**Figure 4:**
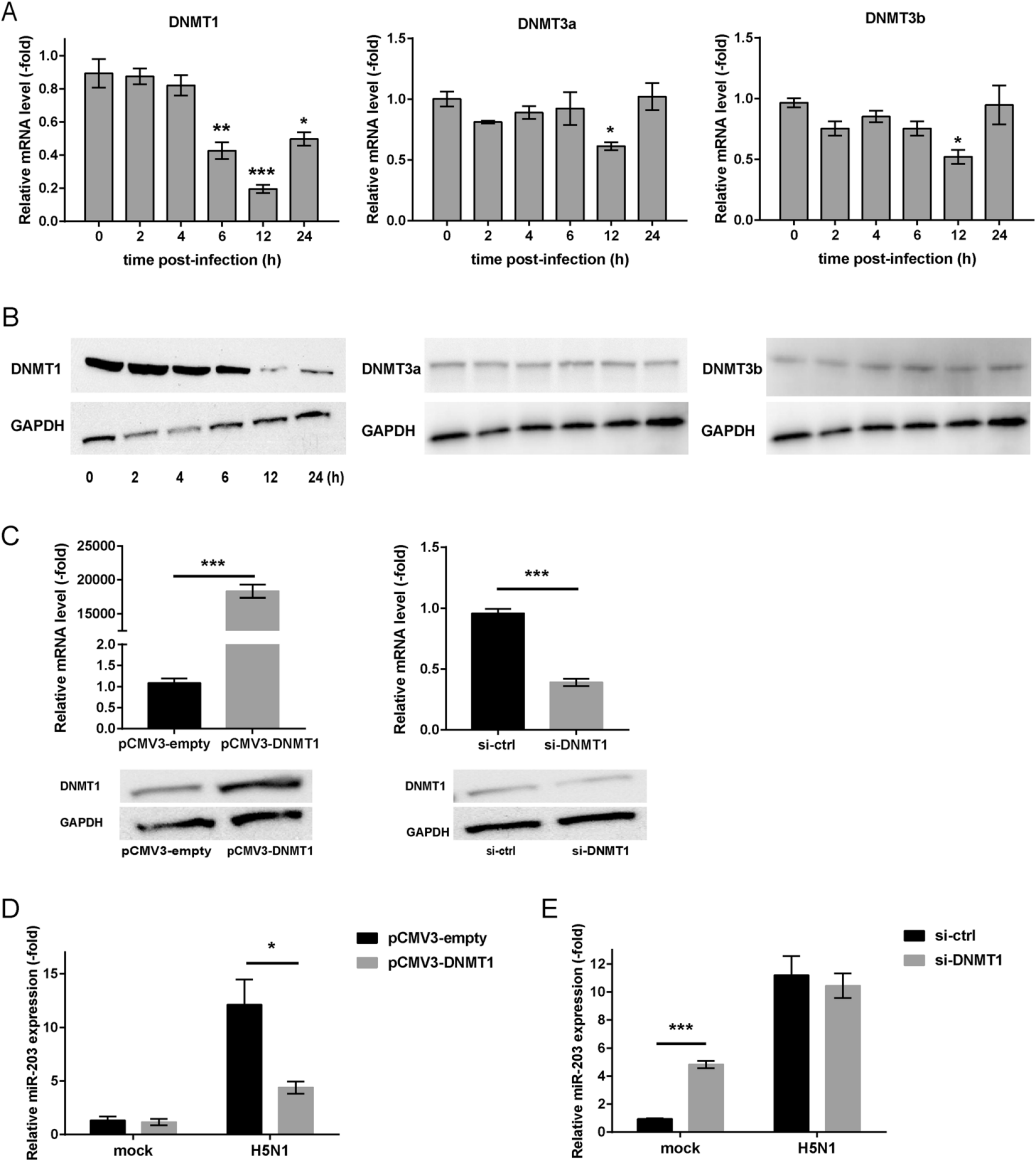
Influenza A virus (IAV) infection inhibits expression of DNA methyltransferase 1 (DNMT1), resulting in up-regulation of miR-203. (**A**) A549 cells were infected with H5N1 (multiplicity of infection (MOI) = 2) viruses, and abundance of mRNAs encoding three DNA methyltransferases (DNMT1, DNMT3a, and DNMT3b) was measured at different times (0, 2, 4, 6, 12, and 24 h). (**B**) Expression of DNMT1, DNMT3a, and DNMT3b protein in A549 cells during H5N1 (MOI = 2) infection was assayed by western blotting. Full-length blots are presented in Supplementary Figures S2–S4. (**C**) A549 cells were transfected for 24 h with pCMV3-DNMT1 or DNMT1-specific siRNA (si-DNMT1). Expression of DNMT1 (pCMV3-DNMT1) and efficiency of si-DNMT1 was measured by qPCR and western blotting. Full-length blots are presented in Supplementary Figure S5. (**D** and **E**) A549 cells were transfected for 24 h with the pCMV3-DNMT1 plasmid (**D**) or si-DNMT1 (**E**). Next, cells were mock-infected or infected with H5N1 (MOI = 2) viruses for another 24 h to detect abundance of miR-203. The empty vector (pCMV3-empty) and si-control served as negative controls. Data are expressed as the mean + SD of three independent experiments. *p < 0.05; **p < 0.01; and ***p < 0.001 (Student’s *t* test).

To obtain more information about DNA demethylation upon IAV infection, we measured expression of three DNA methyltransferases (DNMTs; DNMT1, DNMT3a, and DNMT3b) in A549 cells infected with H5N1 (MOI = 2) viruses at different time points (2, 4, 6, 12, and 24 h) by qPCR. Levels of DNMT1 mRNA fell markedly at 6 h post-infection, while those of DNMT3a and DNMT3b fell only slightly at 12 h post-infection (Fig. 4A). Next, expression of these three DNMTs protein was detected by western blotting; the results were consistent with mRNA levels, showing that DNMT1, but not DNMT3a and DNMT3b, protein was down-regulated obviously at 12 h post-infection (Fig. 4B, Supplementary Figs S2–S4). To further confirm the role of DNMT1, A549 cells were transfected with plasmids expressing DNMT1 (pCMV3-DNMT1) or with DNMT1-specific siRNA (si-DNMT1) (Fig. 4C, Supplementary Fig. S5). At 24 h post-transfection, cells were mock-infected or infected with H5N1 (MOI = 2) viruses for another 24 h. Expression of exogenous DNMT1 protein inhibited expression of miR-203 when compared with cells transfected with empty vectors (Fig. 4D), whereas si-DNMT1 induced production of miR-203 (Fig. 4E). Chiappinelli *et al*. found that DNA methyltransferase inhibitors such as 5-azacytidine and 5-aza-2′-deoxycytidine trigger an interferon response that includes IFN-β, along with expression of a series of IFN-stimulated genes in four ovarian cancer cell lines and one DKO (DNMT1 and DNMT3b knockout) colon cancer cell line^34^. To investigate whether inhibiting DNMT1 affects expression of type I IFNs in A549 cells, we transfected cells with pCMV3-DNMT1 or si-DNMT1, or treated cells with 5-aza-2′-deoxycytidine at a concentration of 1 μM for 48 h, and measured expression of mRNA encoding type I IFNs. We found no change in both IFN-α and IFN-β mRNA expression (Supplementary Fig. S6), suggesting no correlation or at least no direct correlation between DNMTs and expression of type I IFNs in A549 cells. These results show that DNMT1 plays an important role in DNA demethylation during IAV infection, and DNA demethylation could lead to miR-203 expression independently of type I IFNs.

### MiR-203 inhibits IAV replication in A549 cells

**Figure 5:**
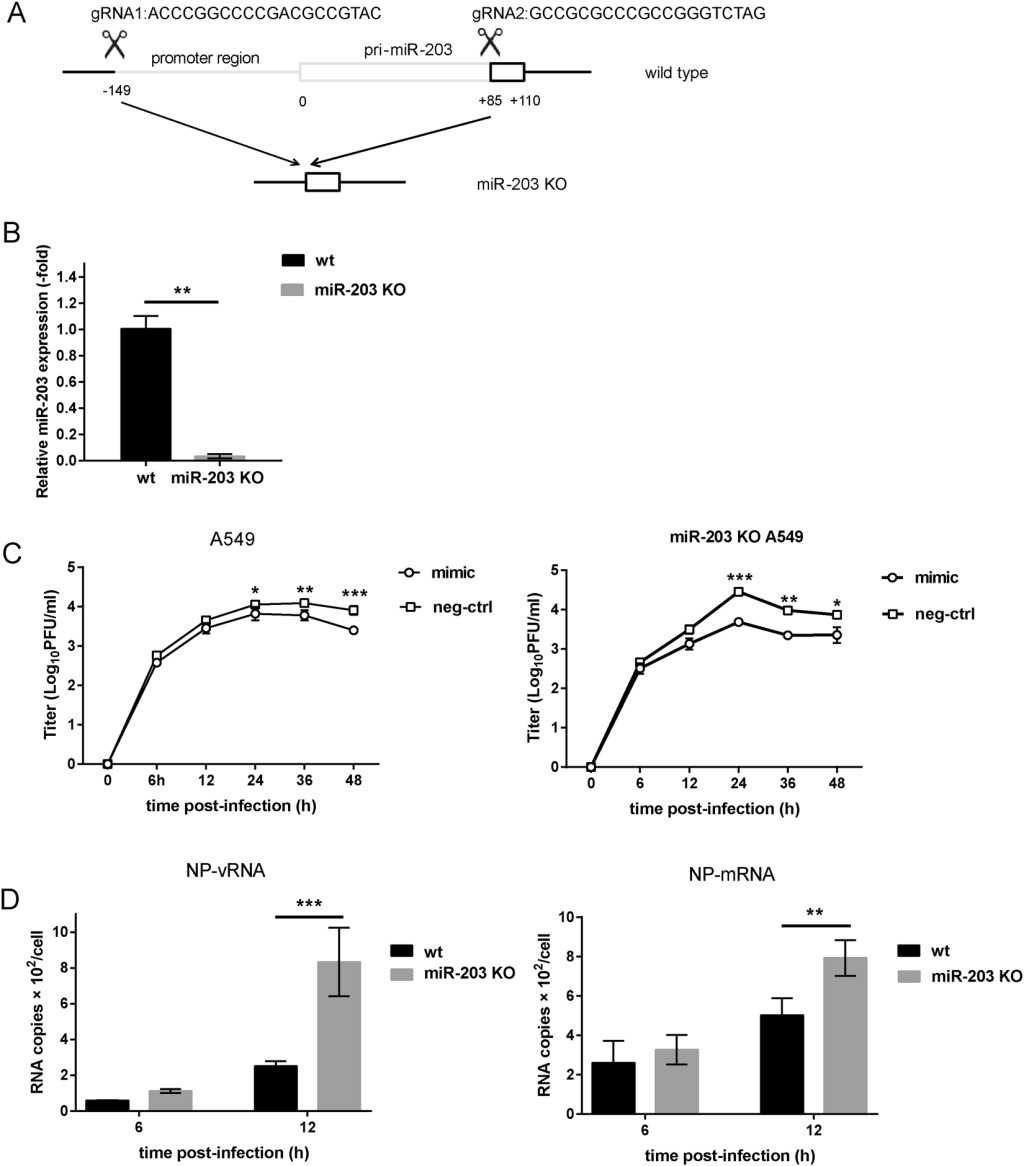
MiR-203 inhibits replication of influenza A virus (IAV) in A549 cells. (**A**) Schematic diagram showing construction of the miR-203 knockout cell line (miR-203 KO cells). The scissors denote the positions of the two gRNAs. (**B**) Biogenesis of mature miR-203 in miR-203 KO cells was assessed by quantitative real-time PCR (qPCR). Data are expressed as the mean + SD of three independent experiments. *p < 0.05; **p < 0.01; and ***p < 0.001 (Student’s *t* test). (**C**) Wild-type A549 cells and miR-203 KO cells were transfected with a miR-203 mimic or a mimic negative control for 24 h. Next, cells were infected with H5N1 (multiplicity of infection (MOI) = 0.01) viruses and virus growth curves were examined. Viral titers were determined in a plaque assay in MDCK cells. (**D**) Wild-type A549 cells and miR-203 KO cells were infected with H5N1 (MOI = 0.01) viruses and harvested at 6 and 12 h post-infection. The copy number of NP vRNA and mRNA was measured in a tag-primed qPCR assay. Data are expressed as the mean + SD of three independent experiments. Statistical significance was calculated using two-way ANOVA (*p < 0.05; **p < 0.01; ***p < 0.001).

To study the effect of miR-203 on IAV replication in more depth, a miR-203 knockout A549 cell line (miR-203 KO cells) was constructed using the CRISPR/CAS9 system. Two gRNAs were designed to target miR-203 (Fig. 5A). The cell line obtained was confirmed by DNA sequencing, which showed that the whole seed region of miR-203, as well as most pri-miR-203 sequences, had been deleted successfully (Fig. 5A). Next, qPCR was performed to examine the biogenesis of mature miR-203. The results showed that miR-203 was almost undetectable in miR-203 KO cells (Fig. 5B). A549 cells and miR-203 KO cells were transfected for 24 h with a miR-203 mimic, followed by infection with H5N1 (MOI = 0.01) virus. Virus growth curves were then generated and examined. The inhibitory effect of miR-203 on IAV replication efficiency was determined in a plaque-forming assay in Madin-Darby canine kidney (MDCK) cells. Virus growth curves revealed that, at 24 h post-infection, fewer IAV particles were released from A549 cells transfected with miR-203 mimic than from the negative control; the results in miR-203 KO cells were consistent with those in wild-type cells (Fig. 5C). Next, we compared the abundance of viral nucleic acids in wild-type and miR-203 KO A549 cells at the early stage of virus infection. The two types of cells were infected with H5N1 (MOI = 0.01) virus and harvested 6 and 12 h later to measure the absolute copy number using a tag-primed qPCR assay. As shown in Fig. 5D, the copy numbers of NP vRNA and mRNA were higher in miR-203 KO cells than in wild-type cells at 12 h post-infection. Taken together, these results show that exogenous expression of miR-203 inhibits IAV replication in A549 cells, and that viral genomic RNA synthesis at the early stage of virus infection is more efficient in miR-203 KO cells.

### DR1 is a novel target of miR-203

**Figure 6:**
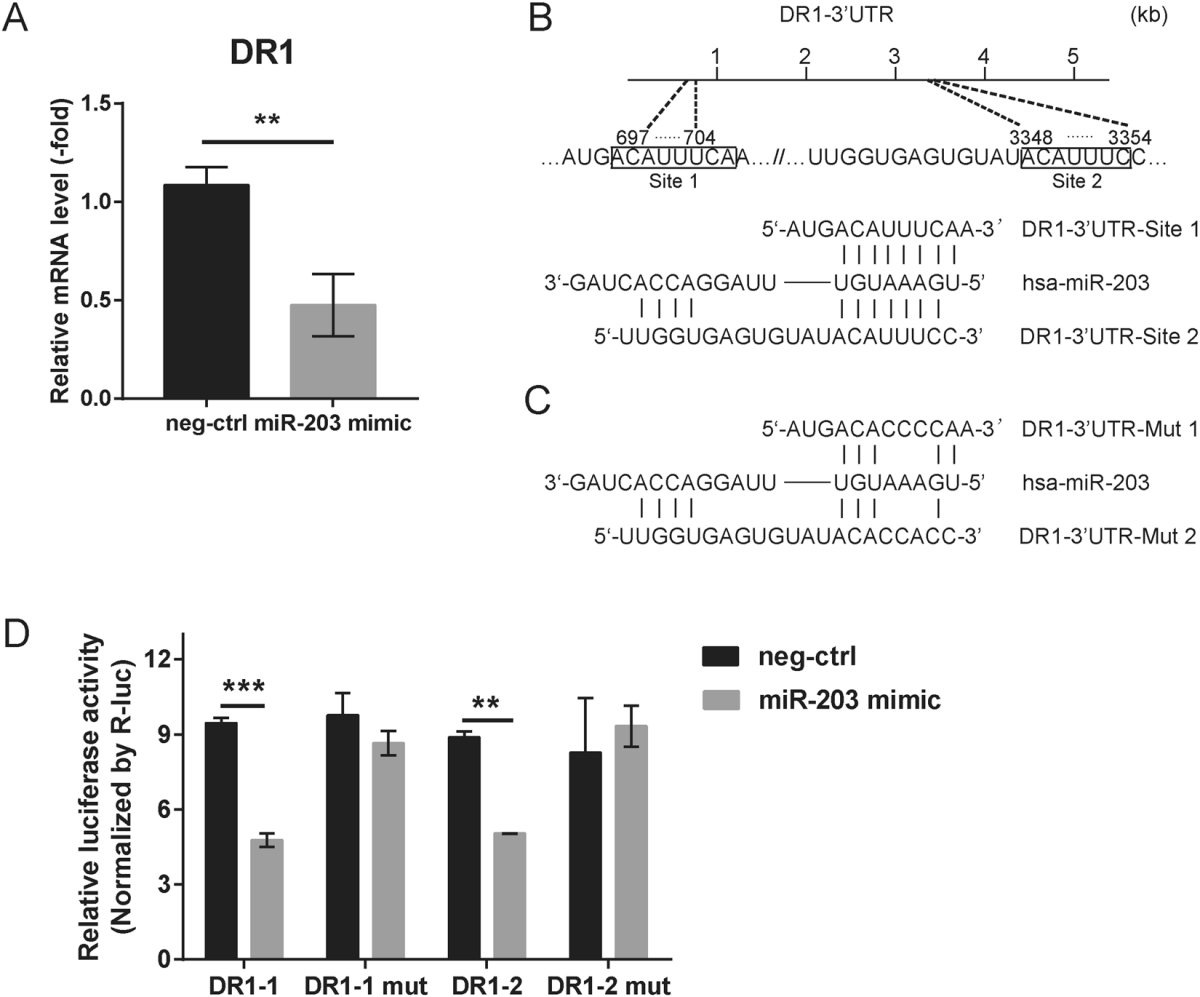
Down-regulator of transcription 1 (DR1) is a novel target of miR-203. (**A**) A549 cells were transfected for 24 h with a miR-203 mimic or a mimic negative control, and abundance of DR1 mRNA was measured by quantitative real-time PCR (qPCR). (**B**) Position of two miR-203-binding sites within the 3′ untranslated region (UTR) of DR1. (**C**) Illustration showing two mutant sequences, DR1-3′UTR-Mut 1 and DR1-3′UTR-Mut 2. (**D**) The luciferase reporter vectors (DR1-3′UTR-Site 1, DR1-3′UTR-Site 2, DR1-3′UTR-Mut 1, and DR1-3′UTR-Mut 2) were transfected into 293 T cells along with a miR-203 mimic. Firefly and Renilla luciferase activity was assayed in a Dual-Luciferase Reporter Assay System 24 h later. Data are expressed as the mean + SD of three independent experiments. *p < 0.05; **p < 0.01; and ***p < 0.001 (Student’s *t* test).

To identify possible target genes for miR-203 during IAV infection, we treated wild-type and miR-203 KO A549 cells with H5N1 (MOI = 2) virus for 48 h. Total RNA was then purified from cells and an Agilent Whole Human Genome Oligo Microarray was performed. The genes showing a 2-fold up-regulation in expression in miR-203 KO cells compared with wild-type A549 cells were selected (Supplementary Fig. S7). Meanwhile, the online tool TargetScanHuman (http://www.targetscan.org/vert_71/) was used to predict biological targets of miR-203. To limit the scope of the prediction, we combined the results provided by two different versions of the TargetScan site and selected genes harboring at least one conserved 8mer site (Supplementary Table S1). By combining predicted genes with the genes differentially expressed in wild-type and miR-203 KO A549 cells, we identified 44 candidate target genes. To further identify one or more novel target genes, we transfected A549 cells for 24 h with the miR-203 mimic and measured the abundance of ten mRNAs (top ten differentially expressed genes) by qPCR. The results revealed a marked down-regulation in DR1 mRNA (Fig. 6A, Supplementary Fig. S8). Sequence analysis showed that DR1 harbors two conserved sites within its 3′ untranslated region (UTR; positions 697–704 and 3348–3354) that match the seed region of miR-203 (Fig. 6B). Next, we sub-cloned the two sites in the DR1 3′UTR (site 1 and site 2) downstream of the firefly luciferase gene within the pmiR-GLO vectors to construct two luciferase reporter vectors (DR1-3′UTR-Site 1 and DR1-3′UTR-Site 2). Also, two mutant plasmids, DR1-3′UTR-Mut 1 and DR1-3′UTR-Mut 2, were constructed by site-directed mutagenesis of the potential target region within miR-203 (Fig. 6C). The luciferase reporter vectors were transfected into 293 T cells along with the miR-203 mimic. Firefly and Renilla luciferase activity was assayed in a Dual-Luciferase Reporter Assay System 24 h after transfection. We found that, in 293 T cells co-transfected with DR1-3′UTR vectors and the miR-203 mimic, the data showed a significant reduction in luciferase activity when compared with that in the control group (co-transfected with DR1-3′UTR vectors plus a mimic negative control). By contrast, the reduction in luciferase reporter activity was completely rescued by mutation of the seed match sequence (Fig. 6D). Taken together, the data show that miR-203 targets DR1 to suppress its expression.

### Silencing of DR1 expression inhibits IAV replication

**Figure 7:**
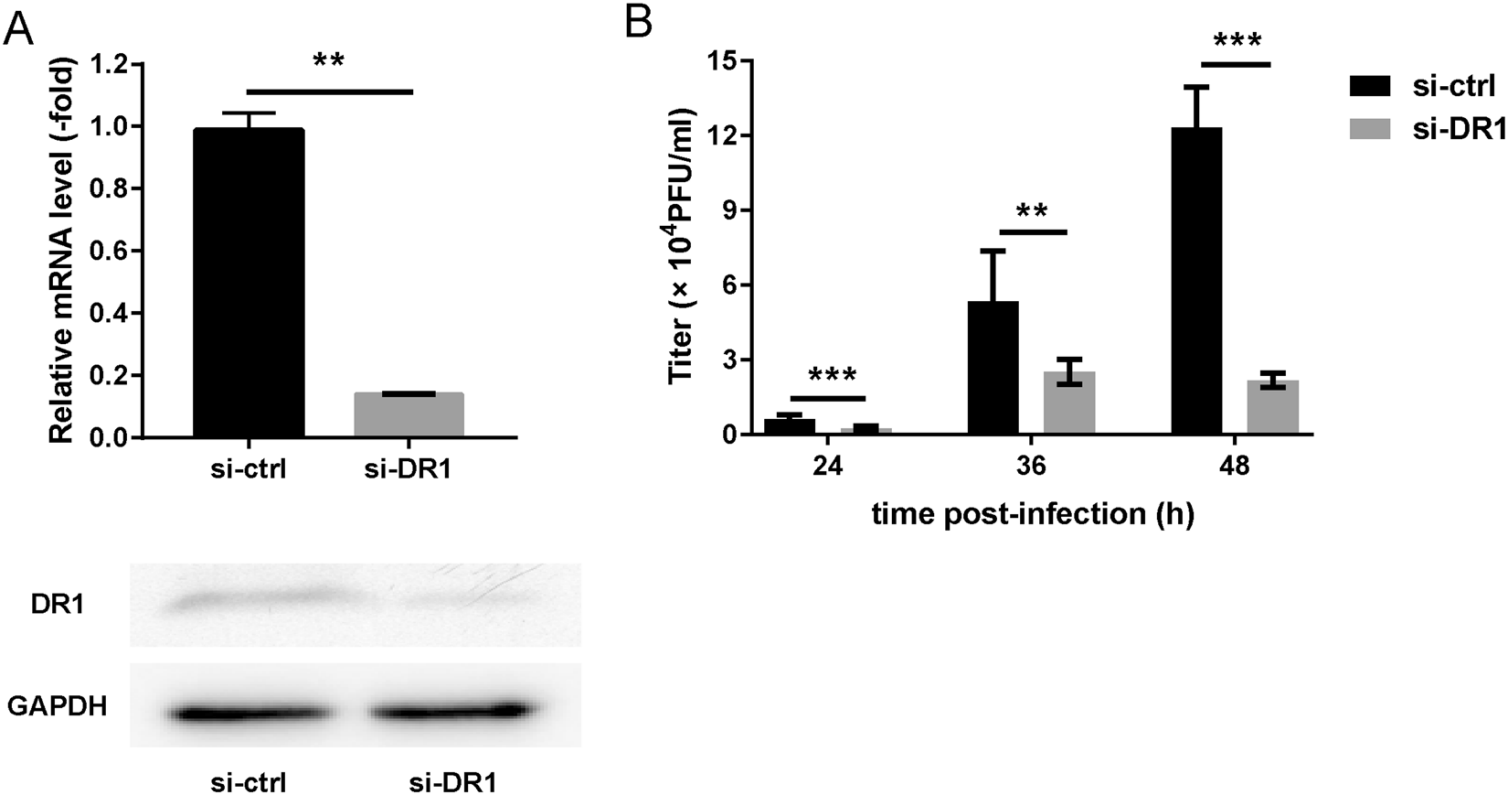
Silencing down-regulator of transcription 1 (DR1) inhibits influenza A virus (IAV) replication. (**A**) A549 cells were transfected with DR1-specific siRNA (si-DR1) for 48 h, and abundance of DR1 was measured by quantitative real-time PCR (qPCR) and western blotting. Full-length blots are presented in Supplementary Figure S9. Data are expressed as the mean + SD of three independent experiments. *p < 0.05; **p < 0.01; ***p < 0.001 (Student’s *t* test). (**B**) miR-203 knockout cells were transfected with si-DR1 or si-control for 48 h, followed by infection with H5N1 (multiplicity of infection = 0.01) viruses. Virus titers were measured in a plaque assay in MDCK cells at 24, 36, and 48 h post-infection. Data are expressed as the mean + SD of three independent experiments. *p < 0.05; **p < 0.01; and ***p < 0.001 (two-way ANOVA).

To examine the effect of down-regulated DR1 expression during IAV infection, miR-203 KO cells were transfected for 48 h with DR1-specific siRNA (si-DR1) or si-control and then infected with H5N1 (MOI = 0.01) virus. Virus titers were measured at 24, 36, and 48 h post-infection. Suppression of DR1 by si-DR1 was verified by qPCR and western blotting (Fig. 7A, Supplementary Fig. S9). The results of the plaque-forming assays showed that virus yields from cells transfected with si-DR1 were lower than in control cells (Fig. 7B). The above data indicate that silencing of DR1 expression inhibits IAV replication. Moreover, miR-203 inhibits IAV replication by targeting DR1.

## Discussion

IAV is a respiratory pathogen that can cause serious illness or even death. Invasion of the virus into host cells leads to complex interactions between the cell and the virus^35–37^. To better elucidate the mechanism underlying regulation of miRNAs and their target genes during IAV infection, we carried out miRNA array analysis of infected A549 cells and identified a series of differentially expressed miRNAs. Among the miRNAs identified, miR-203 was up-regulated after infection by both viruses. Here, we focused on identifying the signaling pathway(s) that regulate miR-203 expression and on the effects of miR-203-mediated feedback regulation on IAV infection.

Type I IFNs play an important role in host innate immune responses to virus infection. Type I IFNs regulate more than 2000 IFN-regulated genes (IRGs), which contain coding and noncoding RNA transcripts, via signaling cascades^38^. Many of these IRGs are miRNAs^27,39–41^. A previous study shows that miR-203 accumulates during Sendai virus mainly via IFN pathway^24^. Therefore, we performed the similar experiments and found that IFN-α induced miR-203 expression directly in both A549 cells and Vero cells, finding consistent with those of the previous study^24^. A dual-luciferase reporter system assay revealed that IFN-α stimulates the promoter region of miR-203. Next, we scanned the 2500 bp promoter region of miR-203 using TRANSFAC and looked for transcription factor-binding sites. Two potential transcription factors associated with the IFN signaling pathways were predicted: ISGF3 and NF-κB. The ISGF3 complex is a key factor involved in the IFN-related JAK-STAT signaling pathway, which comprises signal transducer and activator of transcription 1 (STAT1), STAT2, and IRF9^42^. In addition, the NF-κB pathway is activated by IFN-related signaling cascades^43,44^. These two factors may be instrumental in inducing miR-203 expression and merit further exploration.

Vero cells are IFN-deficient; remarkably, miR-203 was still up-regulated in IAV-infected Vero cells, a finding different from that of Buggele *et al*. They found that miR-203 was not induced by Sendai virus in vero cells^24^. This indicated the mechanisms by which IAV up-regulates miR-203 may be more complicated. Up-regulation of miR-203 in Vero cells (Fig. 2D) indicated that other regulatory mechanisms (in addition to type I IFNs) are also involved in induction of miR-203 during IAV infection.

Epigenetic changes such as DNA methylation are common and important regulators of gene expression^45,46^. Methylation status at gene-regulation positions, particularly in the promoter region, is closely related to the transcriptional status of the gene: hypo-methylation promotes transcription, while hyper-methylation restrains it^47,48^. Jose *et al*. revealed that epigenetic modifications in ALL cell lines (histone modifications and DNA hypermethylation of CpG islands) were important mechanisms involved in regulating expression of miRNAs, including miR-203^30^. Similarly, miR-203 in pancreatic ductal adenocarcinoma^31^, non-small cell lung cancer^32^, and leukemic cell lines^33^ is regulated by DNA methylation of its promoter region. It seems that epigenetic modification is an important and common mechanism underlying regulation of miR-203 expression, particularly during tumorigenesis and tumor progression. Therefore, we measured DNA methylation of the miR-203 promoter regions during IAV infection. Demethylation was detected after IAV infection, leading to miR-203 up-regulation. DNA methylation in mammalian cells is mediated by a complex signaling network, with three DNMTs, DNMT1, DNMT3a, and DNMT3b, playing a major role^46,49^. DNMT1 functions as the major maintenance methyltransferase, while DNMT3a and DNMT3b are required for *de novo* methylation^49^. Here, we found that DNMT1 rather than DNMT3a or DNMT3b was involved in modification of DNA methylation during H5N1 virus infection; this is because expression of DNMT1 was inhibited upon virus infection, whereas that of the other two DNMTs changed little. Therefore, we focused mainly on the function of DNMT1 and found that DNA demethylation in the miR-203 promoter region may be caused by down-regulation of DNMT1 during IAV infection. A previous study demonstrated that the affinity of DNMT1 and DNMT3b for the IL-32 promoter was reduced during IAV infection, resulting in transcriptional activation of IL-32^50^. This may be an important supplement to our research because it supports the notion that decreased affinity of DNMTs for promoter region may also induce expression of miR-203 during IAV infection. However, the underlying signaling pathway through which IAV attenuates DNMT1 expression is still unclear, although the PI3K/AKT/mTOR signaling pathway may suppress expression of DNMT1 and DNMT3a during differentiation of neural stem cells^51^. IAV infection can also activate the PI3K/AKT/mTOR pathway^52–54^, which may be a possible mechanism and requires further experimental verification. However, the precise underlying mechanism may be more complex.

A previous study shows that inhibition of DNA methylation stimulates an IFN response in four ovarian cancer cell lines and one DKO colon cancer cell line^34^. However, we found no correlation between DNMTs and expression of type I IFNs. We speculated that the reasons for the differences may be the different observation period and cell lines. In our study miR-203 was up-regulated at an early stage during IAV infection (Fig. 1D and E). While in research of Chiappinelli *et al*. cells were treated with 5-azacytidine and 5-aza-2′-deoxycytidine for longer duration at 72 h and cells were harvested at 1, 3, or 7 days following initial application of drugs^34^. What’s more, different cell lines used in studies may also lead to different results. As reported previously, the strength of the induced IFN responses was different in the four ovarian cancer cell lines^34^. Thus, we think that type I IFNs are stimulated directly by IAV infection rather than by inhibition of DNA methylation during up-regulation of miR-203. Taken together, both type I IFN and DNA demethylation contributed to induction of miR-203 during IAV infection independently.

The CRISPR/Cas9 system is a gene editing technique that is both specific and stable^55^. Considering the pitfalls inherent in current methods of miRNA silencing using miRNA inhibitors, the use of CRISPR/Cas9 is more efficient^56^. Therefore, we constructed a miR-203 knockout A549 cell line using CRISPR/CAS9 to better explore the effect of miR-203 up-regulation during IAV infection. We found that exogenous expression of miR-203 inhibited IAV replication in both wild-type and miR-203 KO A549 cells. Furthermore, synthesis of IAV viral genomic RNA synthesis at the early stage of infection was more efficient in miR-203 KO cells than in wild-type A549 cells. These results suggest that miR-203 acts as a negative regulator of IAV infection. To identify the target genes involved in inhibiting IAV, we used the Agilent Whole Human Genome Oligo Microarray to analyze genes differentially expressed between IAV-infected wild-type and miR-203 KO A549 cells. The screened genes were then combined with prediction results using the online tool TargetScanHuman. Finally, DR1 was selected as a candidate. DR1 is a cellular corepressor of transcription that coordinates with DRAP1 to assemble a heterodimer called Negative Cofactor 2 (NC2)^57^. NC2 interacts with the TATA-binding protein to preclude RNA polymerase II (Pol II) initiation^58^. Here, we found that DR1 harbors two potentially conserved miR-203-binding sites in the 3′UTR. Exogenous expression of miR-203 suppressed DR1 expression, and a Dual-Luciferase Reporter Assay conducted after co-transfection of a miR-203 mimic plus different luciferase reporter vectors (wild-type or mutant) demonstrated that miR-203 reduced luciferase activity of wild-type reporter vectors but not that of mutant ones. Based on the above-mentioned results, DR1 was identified as a novel target of miR-203.

To determine the effect of down-regulating DR1 expression during IAV infection, we constructed growth curves of IAV viruses in miR-203 KO cells transfected with si-DR1 or si-control. The results showed that si-DR1 suppressed expression of endogenous DR1 and inhibited IAV replication, confirming that miR-203 suppressed IAV proliferation by targeting DR1. DR1 is a host gene that is beneficial for IAV replication because it suppresses IFN induction and interacts with viral RNA-dependent RNA polymerase (RdRp) to directly facilitate viral RNA replication^59^. Our results were consistent with this, further confirming the effect of DR1 on IAV replication.

**Figure 8:**
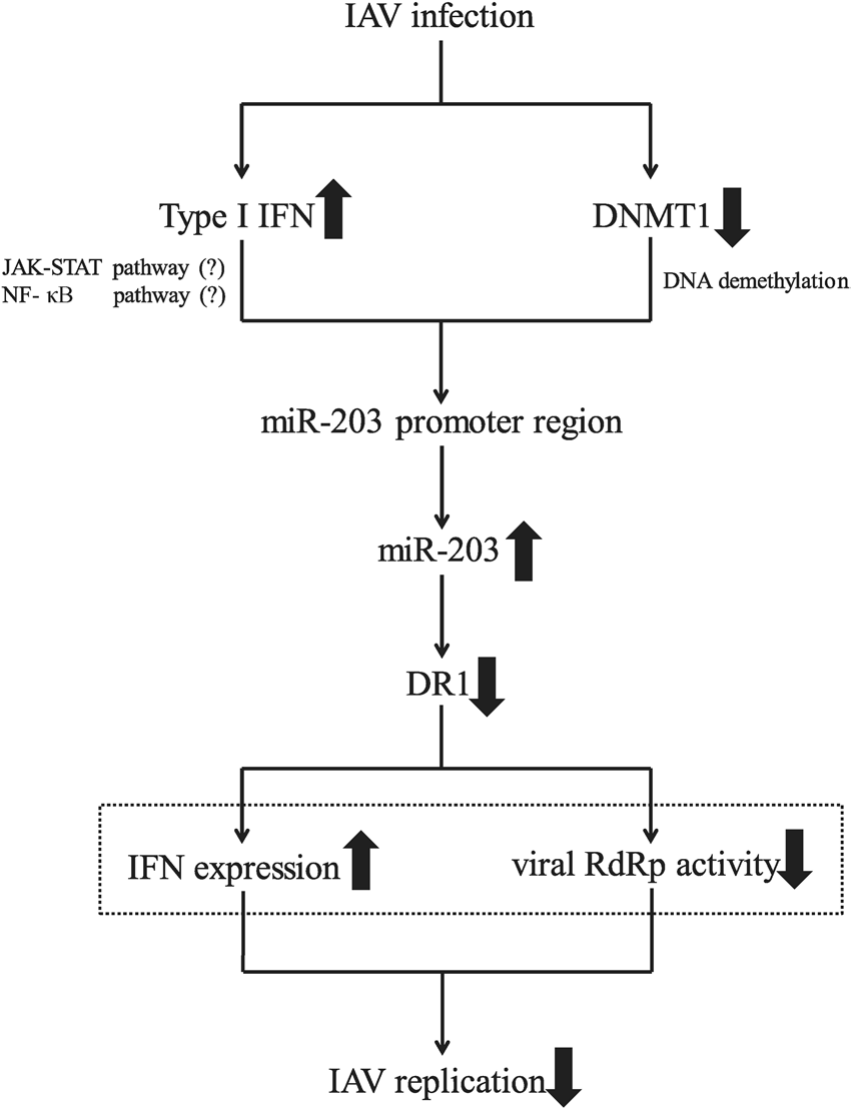
A putative model of the relationship between influenza A virus (IAV) infection and miR-203 expression. IAV infection induces up-regulation of miR-203 via two signaling pathways. Type I interferon (IFN) stimulates the promoter region of miR-203 directly, whereas down-regulation of DNA methyltransferases 1 (DNMT1) leads to DNA demethylation of CpG islands in the miR-203 promoter region. Both of the two processes promote transcription and increase expression of miR-203. MiR-203 suppresses expression of its target gene, down-regulator of transcription 1 (DR1). Suppression of DR1 increases expression of IFN and promotes IAV RNA-dependent RNA polymerase (RdRp) activity, thereby inhibiting IAV replication. The phenomena depicted in the dashed box have been shown by previous studies. The thick arrows indicate up- or down-regulation of gene expression.

In conclusion, we show here that IAV infection of A549 cells up-regulates expression of miR-203. Type I IFN induces transcription of miR-203 directly by stimulating its promoter region. DNA methylation modifications during virus infection also play important roles in this process. Attenuation of DNMT1 expression by IAV leads to demethylation of CpG islands in the miR-203 promoter region, which in turn promotes miR-203 transcription. We also identified DR1, a host gene that plays a positive role in IAV infection, as a novel target of miR-203. In other words, miR-203 targets DR1 to inhibit IAV replication. Based on the above data, we present a signal transduction model that summarizes the signaling pathway that induces expression of miR-203, which then inhibits IAV replication by suppressing its novel target, DR1 (Fig. 8). The results presented herein highlight the potential role of miRNAs as a treatment of IAV infection. To date, antiviral strategies based on miRNA have focused on hepatitis C virus infection^60,61^. Therefore, we hope that this study will provide new ideas for the treatment of IAV or other viral infections.

## Materials and Methods

### Cell culture and viruses

Human lung epithelial cells (A549), human embryonic kidney 293 cells expressing SV40 Large T-antigen (HEK293T), African green monkey kidney cells (Vero), and Madin-Darby canine kidney (MDCK) cells were all purchased from American type culture collection (ATCC, VA, USA) and cryopreserved in the cell repository of our laboratory. They were cultured in Dulbecco’s modified Eagle’s medium (DMEM, Thermo Fisher Scientific, Waltham, MA, USA) supplemented with 10% fetal bovine serum (FBS, PAN-Biotech, Aidenbach, Germany) and 1% penicillin-streptomycin, and maintained at 37 °C in a 5% CO_2_ incubator. A/Vietnam/1194/2004 (H5N1), A/Beijing/501/2009 (H1N1), A/PR/8/34 (H1N1), and A/Anhui/01/2013 (H7N9) viral stocks were propagated in specific pathogen-free (SPF) 10-day-old embryonated eggs, and A/Wisconsin/67/2005 (H3N2) viral stocks were propagated in MDCK cells. All viruses were titrated in a plaque assay on MDCK cells^62^. Harvested viruses were stored at −70 °C until required. Infection of cells by H5N1 virus was performed in DMEM medium supplemented with 2% FBS, while that by H1N1, H3N2, and H7N9 virus was performed in serum-free medium supplemented with 1% bovine serum albumin and 2 μg/ml TPCK-treated trypsin (Sigma-Aldrich, St. Louis, MO, USA).

### Cell treatments

Recombinant human interferon alpha 2 (Hu-IFN-α2; PBL Assay Science, Piscataway, NJ, USA) was added to DMEM medium at a final concentration of 2,000 units/ml. The cell culture medium was replaced with fresh medium containing Hu-IFN-α2 when the cell density reached about 80–90%. Total RNA was extracted from cells after 12 h.

The methylation inhibitor 5-aza-2′-deoxycytidine (Sigma-Aldrich) was used as described previously^63^. The cell culture medium was replaced with fresh medium containing 0.5, 1, or 2 μM 5-aza-2′-deoxycytidine when the cell density reached about 80–90%. The medium was changed every 12 h over a period of 48 h. At the end of treatment, the cell culture medium was replaced with fresh medium without 5-aza-2′-deoxycytidine. Cells were then cultured for another 24 h and used for further experiments. Mock groups were treated under similar conditions but without 5-aza-2′-deoxycytidine.

### Extraction of RNA and DNA and quantitative real-time PCR (qPCR)

For mRNA analysis, total RNA was extracted from cells using a PureLink™ RNA Mini Kit (Thermo Fisher Scientific). QPCR was performed using a One Step SYBR^®^ PrimeScript™ Plus RT-PCR Kit (TaKaRa, Dalian, China P.R.) and specific primer pairs (see Table 1 for sequences). Relative mRNA abundance was calculated by normalizing expression to that of GAPDH using the 2^−ΔΔCt^ method. For miRNA analysis, total RNA was extracted from cells using a mirVana™ miRNA Isolation Kit (Thermo Fisher Scientific). RNA was reverse transcribed using a TaqMan^®^ MicroRNA Reverse Transcription Kit (Thermo Fisher Scientific) and miRNA-specific primers (Thermo Fisher Scientific). QPCR was performed using TaqMan^®^ Universal Master Mix II (Thermo Fisher Scientific) and miRNA-specific probes (Thermo Fisher Scientific). The TaqMan miRNA assays used has-miR-203 and U6 snRNA. Relative miRNA abundance was calculated by normalizing expression to that of U6 snRNA using the 2^−ΔΔCt^ method. Genomic DNA from A549 cells was purified using a PureLink™ Genomic DNA Mini Kit (Thermo Fisher Scientific).

**Table 1:** Sequences of primers and siRNAs used in the study.

Gene name	Forward	Reverse
Primers used for quantitative real-time PCR		
IFN-α	CTTGATGCTCCTGGCACAGA	TCATGGAGGACAGGGATGGT
IFN-β	CATTACCTGAAGGCCAAGGA	CAATTGTCCAGTCCCAGAGG
DNMT1	CCAACAGAGGACAACAAGT	TGGTGGCTGAGTAGTAGAG
DNMT3a	GGACAAGAATGCCACCAA	TCCACCAAGACACAATGC
DNMT3b	TTGGTGATTGGCGGAAG	GAGTAATTCAGCAGGTGGTA
DR1	AGAGTTATTGAGACAGCAACAAGAAT	ATCCCGCCTGATTAGATGC
GAPDH	ACAGCCTCAAGATCATCAG	AGTCCTTCCACGATACCA
Primers used for tag-primed qPCR		
RT-tag-NP-vRNA	GGCCGTCATGGTGGCGAATAGAGAGCAGAAATCCTGG	
RT-tag-NP-mRNA	CCAGATCGTTCGAGTCGTTTTTTTTTTTTTTTTTCTTTAATTTTCATACT	
Forward-NP-vRNA	GGCCGTCATGGTGGCGAAT	
Reverse-NP-vRNA	CTCAAAGTCATATCCACT	
Forward-NP-mRNA	CCAGATCGTTCGAGTCGT	
Reverse-NP-mRNA	CAGAAGATGTGTCATTCCAG	
Primers used for PCR amplification		
Name	Forward	Reverse
miR-203 promoter	ATAATACGCGTCCTTGTCTCTTGCTGTGGTG	ATAATAAGCTTCCCCAACACCGTCGGTTCGC
DR1-3′UTR-Site 1	AGCTTTGTTTAAACGGGTGCTGTGTGAATCAGTAGAC	CTAGTCTAGACCTCTCTCATAGCAAGCATG
DR1-3′UTR-Site 2	AGCTTTGTTTAAACGGATTCATACGCTGGCTATC	CTAGTCTAGAACCCACTAAGATCATTAGCG
DR1-3′UTR-Mut 1	CTATGACACCCCAAAGAC	GTCTTTGGGGTGTCATAG
DR1-3′UTR-Mut 2	CTTTTCCTGAGTGTATACACCACC	GGTGGTGTATACACTCAGGAAAAG
Primers used for PCR prior to DNA methylation analysis		
Sequence no.	Forward	Reverse
No. 1	GGGTGGYTGYAGYAGGGYAGG	RTRRTCCTAAACATTTCACAATTRC
No. 2	GTGGAGGATYAGTYGYGGGAYYTATGG	CACCCCCTRCCCTRCTRCARCCACCC
No. 3	GAAGTGAGAGGGGYTGGGGTGGGTGTG	CCATARRTCCCRCRACTRATCCTCCAC
No. 4	GGTGAYTAAGTGGGTAGGAYYGGYAG	CACACCCACCCCARCCCCTCTCACTTC
No. 5	GATYTGGTGGYTGTGTTYTGGTYTGG	CTRCCRRTCCTACCCACTTARTCACC
No. 6	GAAAAGAAGAGAGGTGGGTTYTTYTTG	CCARACCARAACACARCCACCARATC
No. 7	GGGAGGYYGAGGYGGGTGGATAGYTTG	CAARAARAACCCACCTCTCTTCTTTTC
No. 8	GGAGGTAAYYAGATTTYTYGTGTATG	CAARCTATCCACCCRCCTCRRCCTCCC
No. 9	GTGGTGTGAGGYTGTGGGAGAYTYAG	CATACACRARAAATCTRRTTACCTCC
Sequences of the two gRNAs		
gRNA 1	ACCCGGCCCCGACGCCGTAC	
gRNA 2	GCCGCGCCCGCCGGGTCTAG	
Sequences of the siRNAs		
si-DNMT1	GGCGGCUCAAAGAUUUGGAtt	
si-DR1	GACUCUUCCUAAUGUCCGGtt	

### Tag-primed qPCR assay

A549 monolayers cultured in 24-well plates at 37 °C/5% CO_2_ were infected for 1 h with H5N1 viruses at a MOI of 0.01. Cells were then washed three times with phosphate-buffered saline (PBS) and cultured at 37 °C/5% CO_2_ in DMEM medium supplemented with 2% FBS. The cells were harvested at 6 and 12 h post-infection, and total viral RNA was extracted using a PureLink™ RNA Mini Kit (Thermo Fisher Scientific). Reverse transcription PCR (RT-PCR) was performed using a Transcriptor First Strand cDNA Synthesis Kit (Roche, Basel, Switzerland) and tagged primers (see Table 1 for sequences) to add a specific tag sequence at the 5′ end as previously described^64^. Next, qPCR was performed using SYBR^®^ Premix Ex Taq™ II (TaKaRa) to measure the copy number of mRNA and vRNA encoding NP, with the tagged portion as a forward primer and a segment-specific portion as a reverse primer (see Table 1 for sequences). All experiments were repeated three times. Standard curves were generated by amplifying the target fragments of NP vRNA and mRNA, including the tagged portion, prior to cloning into the pGEM^®^-T Easy Vector (Promega, Madison, WI, USA). Ten-fold serial dilutions of known copy number plasmids were used to generate standard curves.

### Dual-luciferase reporter assay

The 2500 bp genomic sequence (promoter region) upstream of miR-203 was amplified from human genomic DNA (from A549 cells) by PCR and cloned into the pGL3-Basic luciferase reporter vector (Promega). The primers used are shown in the Table 1. A549 cells were co-transfected with the promoter reporter vector and a Renilla luciferase plasmid, pRL-TK (Promega), using Lipofectamine^®^ 3000 (Thermo Fisher Scientific). The cell culture medium was replaced with fresh DMEM medium containing Hu-IFN-α2 at 6 h post-transfection. Mock-transfected cells were not treated with Hu-IFN-α2. Cells were lysed after an additional 24 h, and the luciferase signal was measured using a Dual-Luciferase Reporter Assay System (Promega). The results were expressed as the normalized ratio of Firefly to Renilla luciferase. Genomic DNA from A549 cells was used as a PCR template to amplify 526 and 415 bp sequences of the human DR1 3′ untranslated region (UTR) (see Table 1 for primer sequences). The PCR products were sub-cloned into the pmirGLO dual-luciferase miRNA target expression vector (Promega) to construct luciferase reporter vectors. Meanwhile two mutant plasmids were built by site-directed mutagenesis of the potential miR-203 target region (see Table 1 for primer sequences). 293 T cells were co-transfected with the luciferase reporter vectors and a miR-203 mimic (Thermo Fisher Scientific) using Lipofectamine^®^ 3000 (Thermo Fisher Scientific). Cells were lysed 24 h later, and the luciferase signal was measured as described above. All experiments were repeated three times.

### DNA methylation analysis

The bisulfite conversion and sequencing method was performed to measure methylation of the promoter region within miR-203. Genomic DNA from A549 cells was treated with an EZ DNA Methylation-Direct™ Kit (Zymo Research, Irvine, CA, USA). The optimal amplicon size should be between 150 and 300 bp after bisulfite conversion; therefore, the whole 2500 bp sequence was divided into nine segments. Each segment was amplified separately using specific primer pairs (see Table 1 for sequences), and the PCR products were cloned into pBM23 vector (Biomed, Beijing, China P.R.). Methylation levels were analyzed by sequencing, and the sequencing results of all nine segments were integrated together.

### Western blot analysis and antibodies

Western blot analysis was performed according to a standard protocol^65^. Briefly, whole cells were washed in ice cold PBS before being lysed in RIPA buffer (Thermo Fisher Scientific) containing the protease inhibitor PMSF (Biomed). Next, the proteins in the lysates were separated on SDS-PAGE gels and electrotransferred to PVDF membranes (Millipore, Bedford, MA, USA), followed by blocking in a 5% skim milk solution for 1 h at room temperature under agitation. The membranes were first incubated with primary antibodies and then with HRP-conjugated secondary antibodies. Antibodies were detected using a chemiluminescent HRP substrate (Millipore). Images were obtained using the ImageQuant LAS 500 apparatus (General Electric Healthcare Company, New York, NY, USA). The following antibodies were used: mouse anti-DNMT1 (ab13537, Abcam, Cambridge, UK), rabbit anti-DNMT3a (ab188470, Abcam), rabbit anti-DNMT3b (ab79822, Abcam), mouse anti-GAPDH (ab8245, Abcam), HRP-conjugated rabbit anti-mouse IgG (Sangon Biotech, Shanghai, China P.R.), and HRP-conjugated donkey anti-rabbit IgG (Sangon Biotech).

### Construction of the virus growth curve

A monolayer of A549 cells was incubated for 1 h with H5N1 virus at a MOI of 0.01. Next, cells were washed three times with PBS and cultured at 37 °C/5% CO_2_ in DMEM medium supplemented with 2% FBS. The supernatants were harvested at 6, 12, 24, 36, and 48 h post-infection and stored at −70 °C. Virus titers were measured in a plaque assay on MDCK cells as described previously^62^. All experiments were repeated three times.

### Construction of miR-203 knockout A549 cell lines

Two gRNAs with sequences complementary to miR-203 genes were designed using an online program, CRISPR DESIGN (http://crispr.mit.edu/), developed by Dr. Feng Zhang at MIT (see Table 1 for sequences). The two gRNAs were cloned into a CMV-T7-hspCas9-T2A-GFP-H1-gRNA linearized SmartNuclease vector (CAS740G-1, System Biosciences, Palo Alto, CA, USA) to yield a dual gRNA vector (H1-gRNA1-U6-gRNA2) that targeted miR-203 genomic DNA loci. A549 cells were transfected with the constructed vector using Lipofectamine^®^ 3000 (Thermo Fisher Scientific), and cells expressing GFP were selected by flow cytometry (BD Aria II; Becton, Dickinson and Company, Franklin Lakes, NJ, USA). Single cell clones were cultured in conventional DMEM medium and identified by sequencing and qPCR.

### Plasmids, siRNAs, and other reagents

A human DNMT1 natural ORF mammalian expression plasmid (pCMV3-DNMT1) was purchased from Sino Biological Inc. (HG11494-UT, Shanghai, China P.R.). SiRNAs, including si-DNMT1 and si-DR1, were synthesized by Sangon Biotech (see Table 1 for sequences). Hsa-miR-203a-3p mimic (4464066) and mirVana™ miRNA Mimic Negative Control #1 (4464058) were purchased from Thermo Fisher Scientific.

### MiRNA and mRNA expression profiling analysis

For miRNA expression profiling analysis, A549 cells were infected with H1N1 (MOI = 5) or H5N1 (MOI = 2) viruses for 24 or 48 h. Total RNA was extracted with TRIzol^®^ Reagent (Thermo Fisher Scientific) and miRNA expression profiling analysis was conducted by Gene Square Bio-technology Ltd (Beijing, China P.R.) using a Human v2 MicroRNA Expression Profiling Kit (Illumina, San Diego, CA, USA). The original signal value for each gene point on the chip was calculated and extracted with Illumina GenomeStudio after scanning by BeadStation software. Then the data were normalized (average normalization) using IlluminaGUI in R. For mRNA expression profiling analysis, A549 cells and miR-203 KO A549 cells were infected with H5N1 viruses (MOI = 2) for 48 h and total RNA was extracted with TRIzol^®^ Reagent (Thermo Fisher Scientific). The Agilent Whole Human Genome Oligo Microarray was performed by Kangchen Biotech Inc. (Shanghai, China P.R.). Agilent Feature Extraction software (version 11.0.1.1) was used to analyze acquired array images. Quantile normalization and subsequent data processing were performed with using the GeneSpring GX v12.1 software package (Agilent Technologies). After quantile normalization of the raw data, genes that at least 1 out of all samples have flags in Detected (“All Targets Value”) were chosen for further data analysis. Differentially expressed genes between the two samples were identified through Fold Change filtering.

### Statistical analysis

In all appropriate experiments, *p* values indicating significant differences were evaluated using Student’s *t* test. A *p* value < 0.05 was considered significant. The virus growth curves were analyzed using two-way ANOVA. All graphics and statistical analysis were produced/conducted using GraphPad Prism version 7.00.

### Ethics Statement

The 9-day-old SPF embryonated eggs were purchased from Beijing Merial Vital Laboratory Animal Technology Co. Ltd. (Beijing, China P.R.).

### Data availability

Gene expression data are available from the GEO database (accession IDs GSE107089 and GSE107186). All other relevant data are included in this published article.

## Electronic supplementary material

Supplementary Information
