# Supplementary material for: Up-regulation of microRNA-203 in influenza A virus infection inhibits viral replication by targeting DR1
Source: Sci Rep. 2018 May 1;8:6797. doi: 10.1038/s41598-018-25073-9 (PMC5931597; doi:10.1038/s41598-018-25073-9)
Supplement: Supplementary file 1 — Supplementary Information [file 41598_2018_25073_MOESM1_ESM.pdf]

Up-regulation of microRNA-203 in influenza A virus infection inhibits viral replication by targeting DR1

Sen Zhang, Jing Li, Junfeng Li, Yinhui Yang, Xiaoping Kang, Yuchang Li, Xiaoyan Wu, Qingyu Zhu, Yusen Zhou* & Yi Hu*

State Key Laboratory of Pathogen and Biosecurity, Beijing Institute of Microbiology and Epidemiology, Beijing, 100071, People 's Republic of China.

* Corresponding author

Email: yszhou@bmi.ac.cn (Y.Z.) and huyiamms@163.com (Y.H.)

Supplementary Information includes:

Supplementary Figures S1-S9

Supplementary Table S1

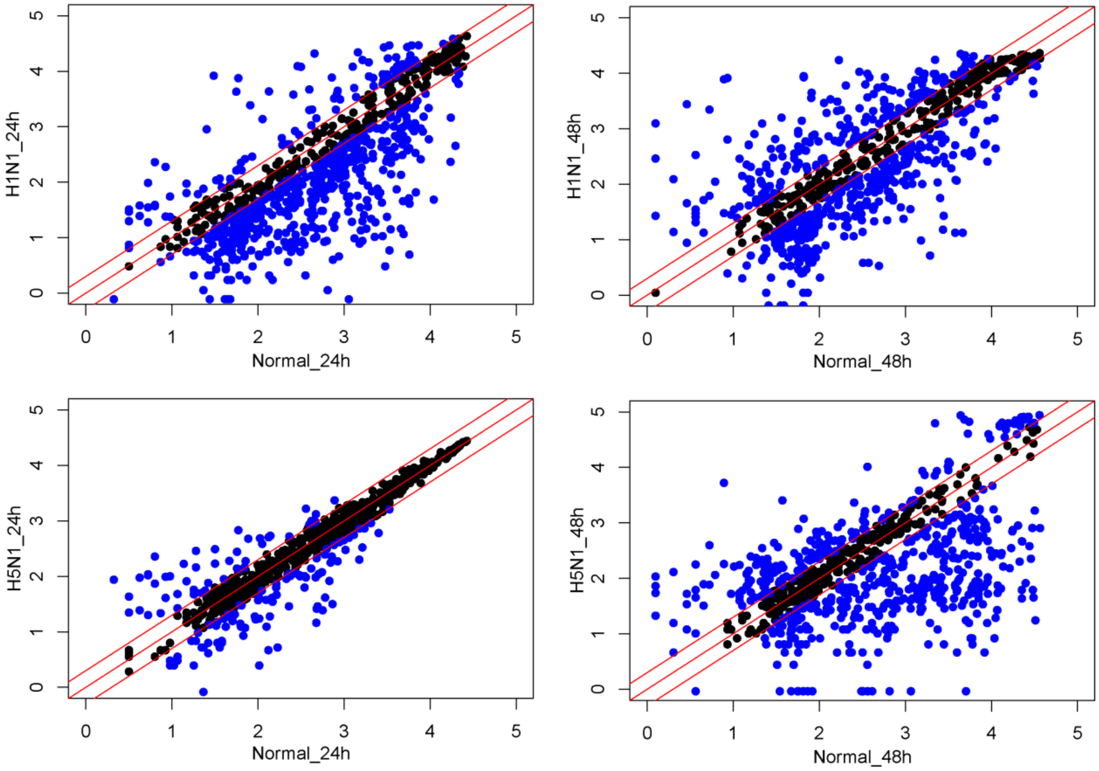

Supplementary Figure S1: Scatter diagrams of the expression level of miRNAs in four groups. The black dots indicate a –fold change in expression < 2, the blue dots in the top left indicate up-regulation, and blue dots in the lower right indicate down-regulation.

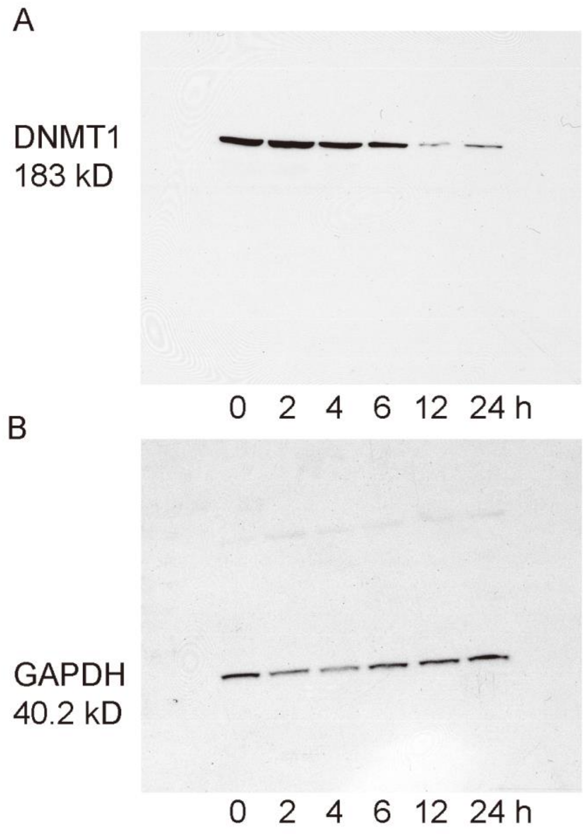

Supplementary Figure S2: Expression of DNMT1 protein expression in A549 cells during H5N1 (MOI=2) infection was assayed by western blotting.

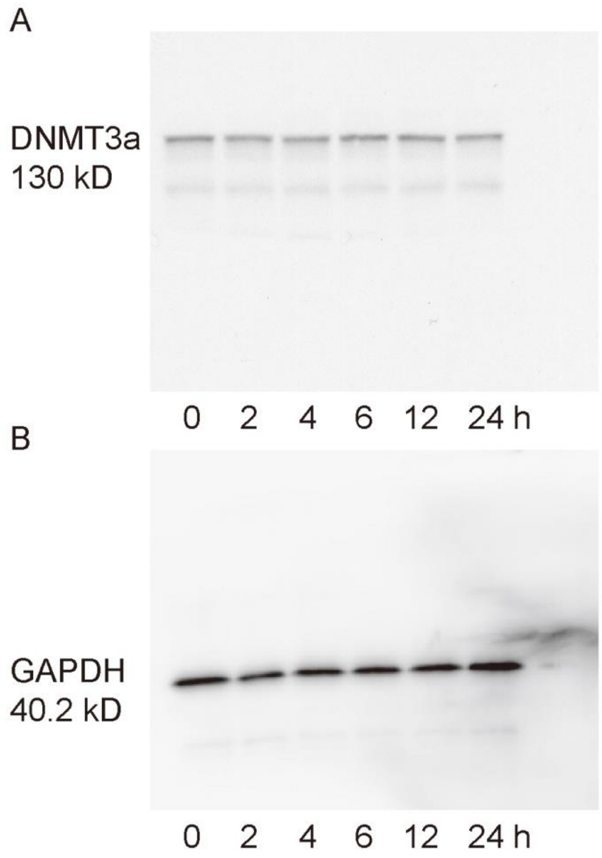

Supplementary Figure S3: Expression of DNMT3a protein expression in A549 cells during H5N1 (MOI=2) infection was assayed by western blotting.

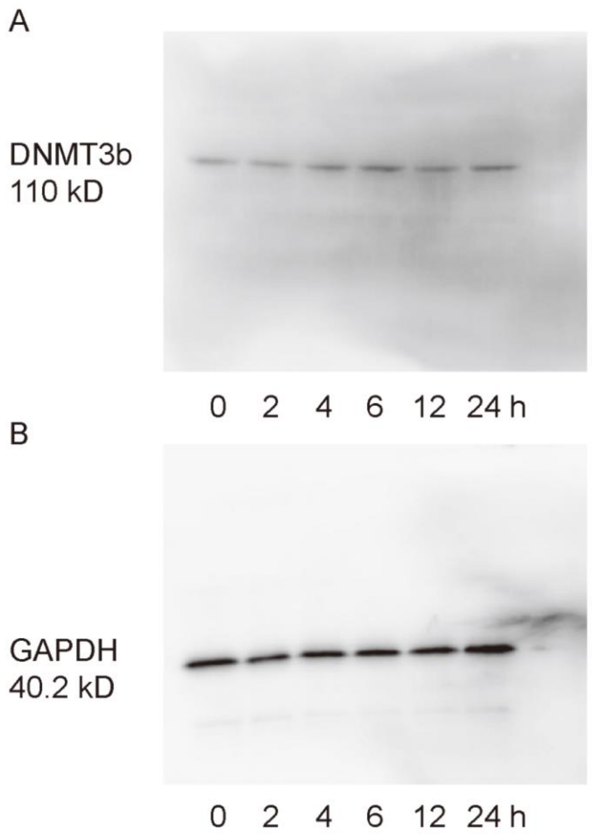

Supplementary Figure S4: Expression of DNMT3b protein expression in A549 cells during H5N1 (MOI=2) infection was assayed by western blotting.

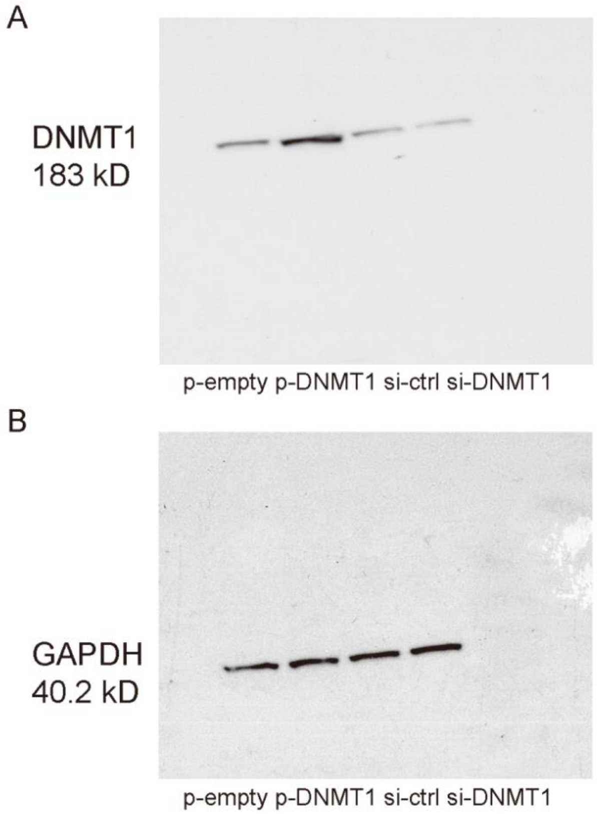

Supplementary Figure S5: Expression of DNMT1 (pCMV3-DNMT1) and efficiency of si-DNMT1 was measured by western blotting.

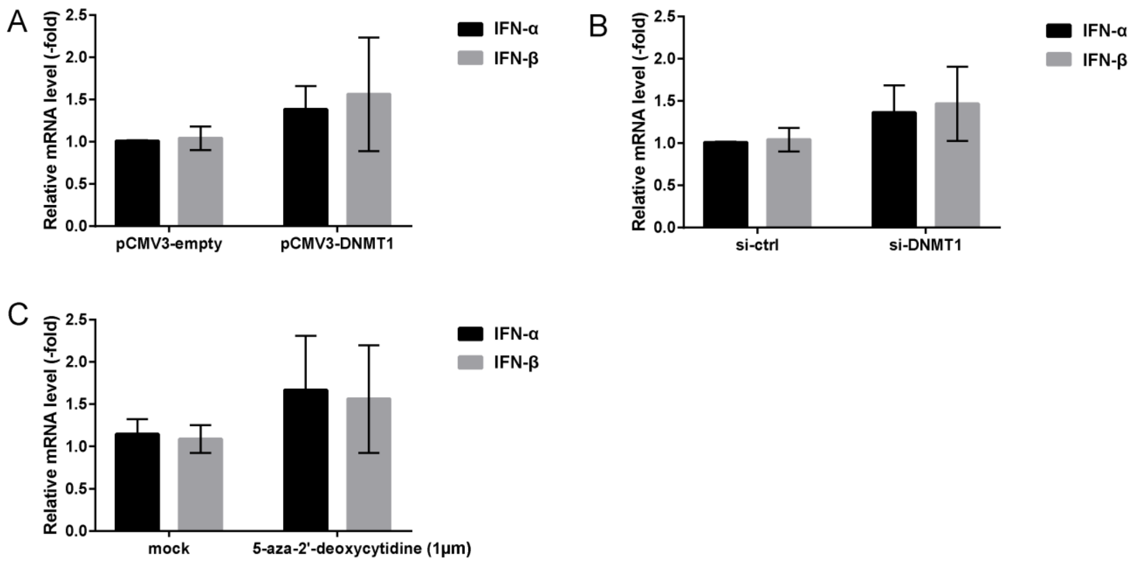

Supplementary Figure S6: (A and B) A549 cells were transfected for 48 h with pCMV3-DNMT1 (A) or DNMT1-specific siRNA (si-DNMT1) (B) to detect mRNA abundance of IFN-α and IFN-β by quantitative real-time PCR (qPCR). The empty vector (pCMV3-empty) and si-control served as negative controls. (C) A549 cells were treated for 48 h with 5-aza-2'-deoxycytidine at a final concentration of 1 μM, and total RNA was purified to analyze mRNA abundance of IFN-α and IFN-β by qPCR. Data are expressed as the mean + SD of three independent experiments. *, $p < 0.05$; **, $p < 0.01$; and *** $p < 0.001$ (Student's t test).

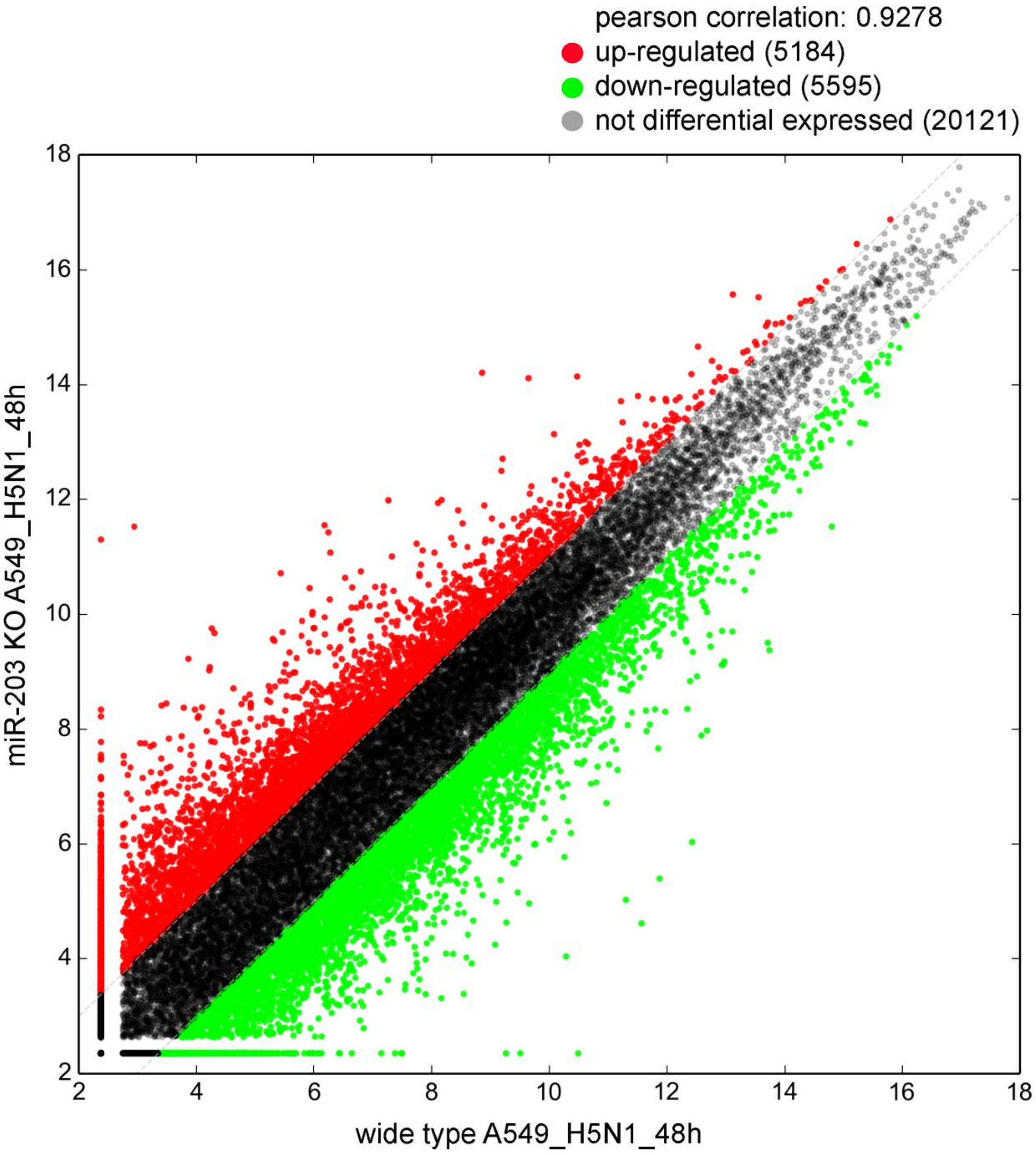

Supplementary Figure S7: Scatter diagram of the expression level of mRNAs in miR-203 knockout cells and wild type A549 cells infected with H5N1 virus (MOI=2). The black dots indicate a –fold change in expression < 2, the red dots indicate up-regulation, and green dots indicate down-regulation.

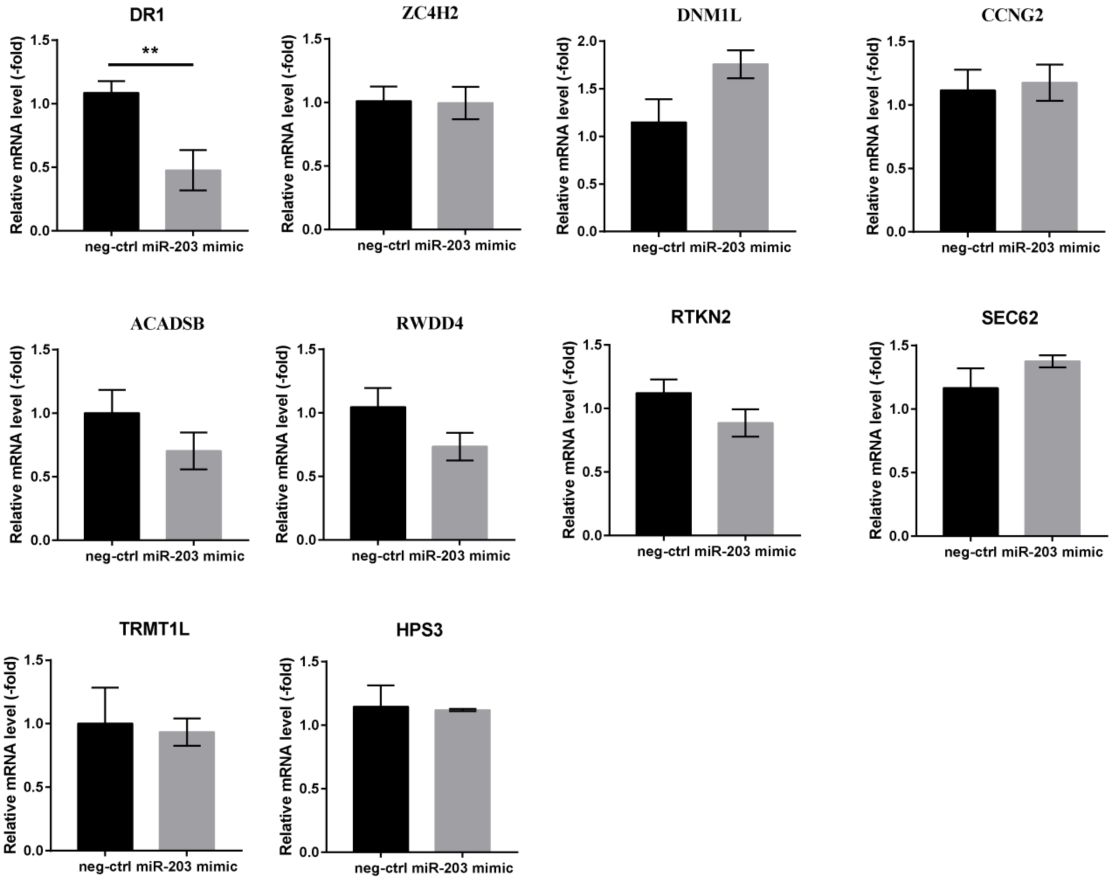

Supplementary Figure S8: A549 cells were transfected for 24 h with the miR-203 mimic and the abundance of 10 mRNA (top 10 differentially expressed genes) were measured by quantitative real-time PCR. Data are expressed as the mean + SD of three independent experiments. *, $p < 0.05$; **, $p < 0.01$; and *** $p < 0.001$ (Student's t test).

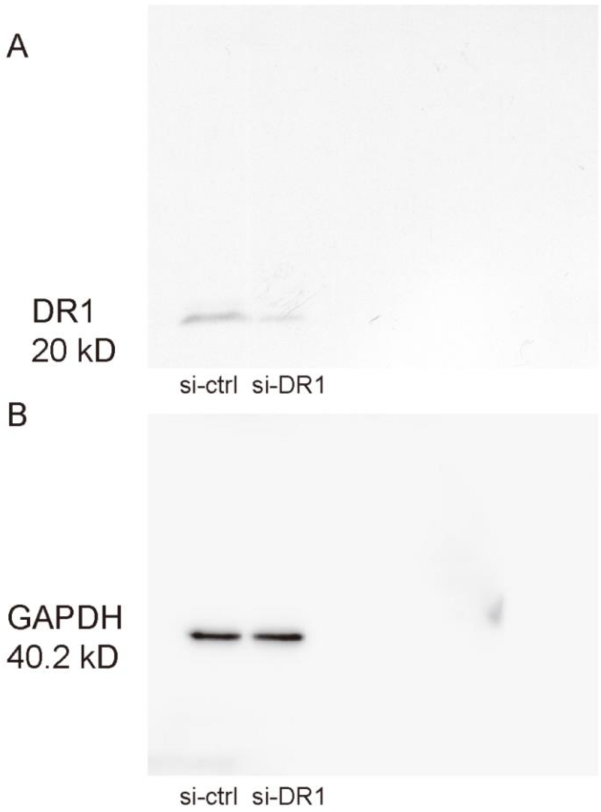

Supplementary Figure S9: Suppression of DR1 by si-DR1 was verified by western blotting.

Supplementary Table S1: Potential target genes of miR-203 predicted by TargetScan.

Ortholog of target gene	Representative transcript	Gene name
LPP	ENST00000312675.4	LIM domain containing preferred translocation partner in lipoma
CAMTA1	ENST00000473578.1	calmodulin binding transcription activator 1
PRPS2	ENST00000380668.5	phosphoribosyl pyrophosphate synthetase 2
ANKRD7	ENST00000357099.4	ankyrin repeat domain 7
LIN7C	ENST00000278193.2	lin-7 homolog C (C. elegans)
C8orf4	ENST00000315792.3	chromosome 8 open reading frame 4
ALG10B	ENST00000308742.4	ALG10B, alpha-1,2-glucosyltransferase
GABRA1	ENST00000428797.2	gamma-aminobutyric acid (GABA) A receptor, alpha 1
SLC4A4	ENST00000340595.3	solute carrier family 4 (sodium bicarbonate cotransporter), member 4
CCNG2	ENST00000316355.5	cyclin G2
FAM126B	ENST00000418596.3	family with sequence similarity 126, member B
TTC39A	ENST00000530004.1	tetratricopeptide repeat domain 39A
ID4	ENST00000378700.3	inhibitor of DNA binding 4, dominant negative helix-loop-helix protein
C4orf33	ENST00000281146.5	chromosome 4 open reading frame 33
SH3BGR	ENST00000380637.3	SH3 domain binding glutamic acid-rich protein
GNG4	ENST00000450593.1	guanine nucleotide binding protein (G protein), gamma 4
PCBP2	ENST00000455667.3	poly(rC) binding protein 2
HNRNPL	ENST00000221419.5	heterogeneous nuclear ribonucleoprotein L
NDRG3	ENST00000373803.2	NDRG family member 3
PARP16	ENST00000261888.6	poly (ADP-ribose) polymerase family, member 16
GPATCH1	ENST00000170564.2	G patch domain containing 1

SOCS3	ENST00000330871.2	suppressor of cytokine signaling 3
RAP2A	ENST00000245304.4	RAP2A, member of RAS oncogene family
PRKAB1	ENST00000229328.5	protein kinase, AMP-activated, beta 1 non-catalytic subunit
MPP3	ENST00000398393.1	membrane protein, palmitoylated 3 (MAGUK p55 subfamily member 3)
TDRD6	ENST00000544460.1	tudor domain containing 6
ADK	ENST00000372734.3	adenosine kinase
TEDDM1	ENST00000367565.1	transmembrane epididymal protein 1
HNRNPUL2	ENST00000301785.5	heterogeneous nuclear ribonucleoprotein U-like 2
GLS	ENST00000338435.4	glutaminase
NCL	ENST00000322723.4	nucleolin
RWDD4	ENST00000326397.5	RWD domain containing 4
EBF3	ENST00000368648.3	early B-cell factor 3
APPL1	ENST00000288266.3	adaptor protein, phosphotyrosine interaction, PH domain and leucine zipper containing 1
COPS7B	ENST00000373608.3	COP9 signalosome subunit 7B
KIF2A	ENST00000381103.2	kinesin heavy chain member 2A
UBE2K	ENST00000261427.5	ubiquitin-conjugating enzyme E2K
HOOK3	ENST00000307602.4	hook microtubule-tethering protein 3
MLANA	ENST00000381477.3	melan-A
HSPB8	ENST00000281938.2	heat shock 22kDa protein 8
ORC2	ENST00000234296.2	origin recognition complex, subunit 2
AKIRIN1	ENST00000432648.3	akirin 1
SEC62	ENST00000337002.4	SEC62 homolog (S. cerevisiae)
CCNG1	ENST00000340828.2	cyclin G1
MBNL3	ENST00000370839.3	muscleblind-like splicing regulator 3
MKL2	ENST00000318282.5	MKL/myocardin-like 2

EBF1	ENST00000313708.6	early B-cell factor 1
SLMO2	ENST00000355937.4	slowmo homolog 2 (Drosophila)
SLC30A6	ENST00000282587.5	solute carrier family 30 (zinc transporter), member 6
ZFHX4	ENST00000521891.2	zinc finger homeobox 4
RUNX2	ENST00000371432.3	runt-related transcription factor 2
DUSP5	ENST00000369583.3	dual specificity phosphatase 5
CUL1	ENST00000325222.4	cullin 1
KIAA1429	ENST00000437199.1	KIAA1429
DOCK10	ENST00000409592.3	dedicator of cytokinesis 10
ZFP1	ENST00000464850.1	ZFP1 zinc finger protein
PACS2	ENST00000447393.1	phosphofurin acidic cluster sorting protein 2
ANTXR2	ENST00000403729.2	anthrax toxin receptor 2
NAA30	ENST00000556492.1	N(alpha)-acetyltransferase 30, NatC catalytic subunit
ZMYM2	ENST00000382869.3	zinc finger, MYM-type 2
ABCE1	ENST00000296577.4	ATP-binding cassette, sub-family E (OABP), member 1
CITED2	ENST00000367651.2	Cbp/p300-interacting transactivator, with Glu/Asp-rich carboxy-terminal domain, 2
PAQR3	ENST00000512733.1	progestin and adipoQ receptor family member III
GPR85	ENST00000297146.3	G protein-coupled receptor 85
RALB	ENST00000272519.5	v-ral simian leukemia viral oncogene homolog B
SRSF1	ENST00000258962.4	serine/arginine-rich splicing factor 1
DNAJC21	ENST00000382021.2	DnaJ (Hsp40) homolog, subfamily C, member 21
CREBZF	ENST00000398294.2	CREB/ATF bZIP transcription factor
TWF1	ENST00000552521.1	twinfilin actin-binding protein 1
OVOL1	ENST00000335987.3	ovo-like 1(Drosophila)
CNNM3	ENST00000377060.3	cyclin M3
AFAP1L2	ENST00000304129.4	actin filament associated protein 1-like 2

UCHL5	ENST00000367455.4	ubiquitin carboxyl-terminal hydrolase L5
KRT1	ENST00000252244.3	keratin 1
BMI1	ENST00000376663.3	BMI1 polycomb ring finger oncogene
TOX3	ENST00000407228.3	TOX high mobility group box family member 3
LNX2	ENST00000316334.3	ligand of numb-protein X 2
NEDD4L	ENST00000256832.7	neural precursor cell expressed, developmentally down-regulated 4-like, E3 ubiquitin protein ligase
KRT85	ENST00000257901.3	keratin 85
SGTB	ENST00000381007.4	small glutamine-rich tetratricopeptide repeat (TPR)-containing, beta
PRKCB	ENST00000303531.7	protein kinase C, beta
KLHL15	ENST00000328046.8	kelch-like family member 15
PAPSS2	ENST00000361175.4	3'-phosphoadenosine 5'-phosphosulfate synthase 2
TADA1	ENST00000367874.4	transcriptional adaptor 1
SGMS2	ENST00000394684.4	sphingomyelin synthase 2
AFF4	ENST00000265343.5	AF4/FMR2 family, member 4
CPEB4	ENST00000265085.5	cytoplasmic polyadenylation element binding protein 4
ARHGAP42	ENST00000524892.2	Rho GTPase activating protein 42
TMOD2	ENST00000249700.4	tropomodulin 2 (neuronal)
SESTD1	ENST00000428443.3	SEC14 and spectrin domains 1
EIF5A2	ENST00000474096.1	eukaryotic translation initiation factor 5A2
TGFB2	ENST00000366930.4	transforming growth factor, beta 2
COMMD3-BMI1	ENST00000602390.1	COMMD3-BMI1 readthrough
TLL2	ENST00000357947.3	tolloid-like 2
SEMA5A	ENST00000382496.5	sema domain, seven thrombospondin repeats (type 1 and type 1-like), transmembrane domain (TM) and short cytoplasmic domain, (semaphorin) 5A

TCF4	ENST00000354452.3	transcription factor 4
GXYLT1	ENST00000398675.3	glucoside xylosyltransferase 1
AHR	ENST00000242057.4	aryl hydrocarbon receptor
DCP2	ENST00000389063.2	decapping mRNA 2
ZMYND11	ENST00000381591.1	zinc finger, MYND-type containing 11
ZC4H2	ENST00000545618.1	zinc finger, C4H2 domain containing
UPF2	ENST00000356352.2	UPF2 regulator of nonsense transcripts homolog (yeast)
TADA2B	ENST00000310074.7	transcriptional adaptor 2B
UBP1	ENST00000283629.3	upstream binding protein 1 (LBP-1a)
CXorf23	ENST00000379687.3	chromosome X open reading frame 23
KIAA1009	ENST00000257766.4	KIAA1009
EGR3	ENST00000317216.2	early growth response 3
RAB27B	ENST00000262094.5	RAB27B, member RAS oncogene family
TRMT1L	ENST00000367504.3	tRNA methyltransferase 1 homolog (S. cerevisiae)-like
OPA1	ENST00000392438.3	optic atrophy 1 (autosomal dominant)
NFYA	ENST00000341376.6	nuclear transcription factor Y, alpha
GMFB	ENST00000358056.3	glia maturation factor, beta
IRS2	ENST00000375856.3	insulin receptor substrate 2
ZNF292	ENST00000369577.3	zinc finger protein 292
PRICKLE2	ENST00000295902.6	prickle homolog 2 (Drosophila)
NCALD	ENST00000395923.1	neurocalcin delta
SLC39A9	ENST00000031146.4	solute carrier family 39, member 9
APC	ENST00000457016.1	adenomatous polyposis coli
PAPD5	ENST00000357464.3	PAP associated domain containing 5
AAK1	ENST00000409085.4	AP2 associated kinase 1
DR1	ENST00000370272.4	down-regulator of transcription 1, TBP-binding (negative cofactor 2)

NEDD9	ENST00000379446.5	neural precursor cell expressed, developmentally down-regulated 9
CHD9	ENST00000566029.1	chromodomain helicase DNA binding protein 9
TRIM71	ENST00000383763.5	tripartite motif containing 71, E3 ubiquitin protein ligase
SLC7A14	ENST00000231706.5	solute carrier family 7, member 14
MYO5A	ENST00000399231.3	myosin VA (heavy chain 12, myoxin)
NPR3	ENST00000265074.8	natriuretic peptide receptor C/guanylate cyclase C (atrionatriuretic peptide receptor C)
KHDRBS1	ENST00000327300.7	KH domain containing, RNA binding, signal transduction associated 1
PDGFD	ENST00000393158.2	platelet derived growth factor D
LASP1	ENST00000318008.6	LIM and SH3 protein 1
NR1D2	ENST00000312521.4	nuclear receptor subfamily 1, group D, member 2
STRN	ENST00000263918.4	striatin, calmodulin binding protein
RASAL2	ENST00000448150.3	RAS protein activator like 2
DLG5	ENST00000372391.2	discs, large homolog 5 (Drosophila)
PLEKHG3	ENST00000247226.7	pleckstrin homology domain containing, family G (with RhoGef domain) member 3
C12orf4	ENST00000261250.3	chromosome 12 open reading frame 4
ANKRD13C	ENST00000370944.4	ankyrin repeat domain 13C
DGKB	ENST00000403951.2	diacylglycerol kinase, beta 90kDa
LPIN1	ENST00000256720.2	lipin 1
MAP3K1	ENST00000399503.3	mitogen-activated protein kinase kinase kinase 1, E3 ubiquitin protein ligase
CDH6	ENST00000265071.2	cadherin 6, type 2, K-cadherin (fetal kidney)
FUBP3	ENST00000319725.9	far upstream element (FUSE) binding protein 3
BPTF	ENST00000321892.4	bromodomain PHD finger transcription factor

OSBPL8	ENST00000393249.2	oxysterol binding protein-like 8
CREB3	ENST00000353704.2	cAMP responsive element binding protein 3
PRKCI	ENST00000295797.4	protein kinase C, iota
FAM155A	ENST00000375915.2	family with sequence similarity 155, member A
PXDN	ENST00000252804.4	peroxidasin homolog (Drosophila)
MYEF2	ENST00000324324.7	myelin expression factor 2
JOSD1	ENST00000216039.5	Josephin domain containing 1
RAPGEF1	ENST00000372189.3	Rap guanine nucleotide exchange factor (GEF) 1
FOSL2	ENST00000379619.1	FOS-like antigen 2
PLAA	ENST00000397292.3	phospholipase A2-activating protein
KCNK10	ENST00000340700.5	potassium channel, subfamily K, member 10
RLIM	ENST00000332687.6	ring finger protein, LIM domain interacting
ARID2	ENST00000457135.1	AT rich interactive domain 2 (ARID, RFX-like)
SRC	ENST00000373578.2	v-src avian sarcoma (Schmidt-Ruppin A-2) viral oncogene homolog
RBMS1	ENST00000348849.3	RNA binding motif, single stranded interacting protein 1
ZEB1	ENST00000361642.5	zinc finger E-box binding homeobox 1
ROBO2	ENST00000461745.1	roundabout, axon guidance receptor, homolog 2 (Drosophila)
ZBTB46	ENST00000245663.4	zinc finger and BTB domain containing 46
ZNF532	ENST00000336078.4	zinc finger protein 532
PHF12	ENST00000577226.1	PHD finger protein 12
FAM117B	ENST00000392238.2	family with sequence similarity 117, member B
KIAA1211	ENST00000504228.1	KIAA1211
GABARAPL1	ENST00000266458.5	GABA(A) receptor-associated protein like 1
KCMF1	ENST00000409785.4	potassium channel modulatory factor 1
ATG14	ENST00000247178.5	autophagy related 14

MED14	ENST00000324817.1	mediator complex subunit 14
RSBN1	ENST00000261441.5	round spermatid basic protein 1
ESR1	ENST00000440973.1	estrogen receptor 1
MEGF11	ENST00000360698.4	multiple EGF-like-domains 11
SPEN	ENST00000375759.3	spen homolog, transcriptional regulator (Drosophila)
CHD2	ENST00000394196.4	chromodomain helicase DNA binding protein 2
GABBR2	ENST00000259455.2	gamma-aminobutyric acid (GABA) B receptor, 2
SPATA13	ENST00000382108.3	spermatogenesis associated 13
SLC12A2	ENST00000262461.2	solute carrier family 12 (sodium/potassium/chloride transporter), member 2
ZCCHC14	ENST00000268616.4	zinc finger, CCHC domain containing 14
VCPIP1	ENST00000310421.4	valosin containing protein (p97)/p47 complex interacting protein 1
ADAMTS5	ENST00000284987.5	ADAM metallopeptidase with thrombospondin type 1 motif, 5
CORO1C	ENST00000261401.3	coronin, actin binding protein, 1C
CDK13	ENST00000181839.4	cyclin-dependent kinase 13
SPATA5	ENST00000274008.4	spermatogenesis associated 5
DNM1L	ENST00000452533.2	dynamin 1-like
ZNRF3	ENST00000544604.2	zinc and ring finger 3
ZC3H12D	ENST00000416573.2	zinc finger CCCH-type containing 12D
FOXP1	ENST00000318789.4	forkhead box P1
ASXL1	ENST00000375687.4	additional sex combs like 1 (Drosophila)
VGLL4	ENST00000273038.3	vestigial like 4 (Drosophila)
GPR155	ENST00000392552.2	G protein-coupled receptor 155
ACADSB	ENST00000358776.4	acyl-CoA dehydrogenase, short/branched chain
KYNU	ENST00000264170.4	kynureninase

C14orf28	ENST00000325192.3	chromosome 14 open reading frame 28
WAC	ENST00000375664.4	WW domain containing adaptor with coiled-coil
NKD1	ENST00000268459.3	naked cuticle homolog 1 (Drosophila)
HPS3	ENST00000296051.2	Hermansky-Pudlak syndrome 3
SMURF1	ENST00000361368.2	SMAD specific E3 ubiquitin protein ligase 1
USP15	ENST00000353364.3	ubiquitin specific peptidase 15
DCUN1D5	ENST00000260247.5	DCN1, defective in cullin neddylation 1, domain containing 5
VWA3B	ENST00000477737.1	von Willebrand factor A domain containing 3B
ATP1B4	ENST00000218008.3	ATPase, Na+/K+ transporting, beta 4 polypeptide
CAPS	ENST00000222125.5	calcyphosine
ADPGK	ENST00000311669.8	ADP-dependent glucokinase
CRK	ENST00000398970.5	v-crk avian sarcoma virus CT10 oncogene homolog
MAP3K2	ENST00000409947.1	mitogen-activated protein kinase kinase kinase 2
RTKN2	ENST00000373789.3	rhotekin 2
ZNF148	ENST00000360647.4	zinc finger protein 148
PPP1R12A	ENST00000261207.5	protein phosphatase 1, regulatory subunit 12A
SOCS6	ENST00000397942.3	suppressor of cytokine signaling 6
PLD1	ENST00000342215.6	phospholipase D1, phosphatidylcholine-specific
PDE4D	ENST00000340635.6	phosphodiesterase 4D, cAMP-specific
SP4	ENST00000222584.3	Sp4 transcription factor
TRPS1	ENST00000395715.3	trichorhinophalangeal syndrome I
SYNCRIP	ENST00000355238.6	synaptotagmin binding, cytoplasmic RNA interacting protein
SEL1L	ENST00000336735.4	sel-1 suppressor of lin-12-like (C. elegans)
COL4A4	ENST00000396625.3	collagen, type IV, alpha 4
TRPV3	ENST00000301365.4	transient receptor potential cation channel, subfamily V,

		member 3
ELK4	ENST00000357992.4	ELK4, ETS-domain protein (SRF accessory protein 1)
NUFIP2	ENST00000225388.4	nuclear fragile X mental retardation protein interacting protein 2
ZNF281	ENST00000294740.3	zinc finger protein 281
